# Competing Mechanistic Hypotheses of Acetaminophen-Induced Hepatotoxicity Challenged by Virtual Experiments

**DOI:** 10.1371/journal.pcbi.1005253

**Published:** 2016-12-16

**Authors:** Andrew K. Smith, Brenden K. Petersen, Glen E. P. Ropella, Ryan C. Kennedy, Neil Kaplowitz, Murad Ookhtens, C. Anthony Hunt

**Affiliations:** 1 Bioengineering and Therapeutic Sciences, University of California, San Francisco, San Francisco, CA, United States of America; 2 UCSF/UCB Joint Graduate Group in Bioengineering, University of California, Berkeley, Berkeley, CA, United States of America; 3 Tempus Dictum, Inc., Milwaukie, OR, United States of America; 4 Division of Gastrointestinal and Liver Diseases, Department of Medicine, Keck School of Medicine, University of Southern California, Los Angeles, CA, United States of America; Johns Hopkins University, UNITED STATES

## Abstract

Acetaminophen-induced liver injury in mice is a model for drug-induced liver injury in humans. A precondition for improved strategies to disrupt and/or reverse the damage is a credible explanatory mechanism for how toxicity phenomena emerge and converge to cause hepatic necrosis. The Target Phenomenon in mice is that necrosis begins adjacent to the lobule’s central vein (CV) and progresses outward. An explanatory mechanism remains elusive. Evidence supports that location dependent differences in NAPQI (the reactive metabolite) formation within hepatic lobules (NAPQI zonation) are necessary and sufficient prerequisites to account for that phenomenon. We call that the NZ-mechanism hypothesis. Challenging that hypothesis in mice is infeasible because 1) influential variables cannot be controlled, and 2) it would require sequential intracellular measurements at different lobular locations within the same mouse. Virtual hepatocytes use independently configured periportal-to-CV gradients to exhibit lobule-location dependent behaviors. Employing NZ-mechanism achieved quantitative validation targets for acetaminophen clearance and metabolism but failed to achieve the Target Phenomenon. We posited that, in order to do so, at least one additional feature must exhibit zonation by decreasing in the CV direction. We instantiated and explored two alternatives: 1) a glutathione depletion threshold diminishes in the CV direction; and 2) ability to repair mitochondrial damage diminishes in the CV direction. Inclusion of one or the other feature into NZ-mechanism failed to achieve the Target Phenomenon. However, inclusion of both features enabled successfully achieving the Target Phenomenon. The merged mechanism provides a multilevel, multiscale causal explanation of key temporal features of acetaminophen hepatotoxicity in mice. We discovered that variants of the merged mechanism provide plausible quantitative explanations for the considerable variation in 24-hour necrosis scores among 37 genetically diverse mouse strains following a single toxic acetaminophen dose.

## Author Summary

Acetaminophen-induced liver injury in mice is a model for drug-induced liver injury in humans. Challenging an explanatory mechanism in mice is problematic because variables determining causes and effects cannot be controlled adequately. We circumvent that impediment by performing virtual experiments that challenge the prevailing scientific explanation for the characteristic spatiotemporal pattern of early acetaminophen-induced hepatic necrosis. Virtual mice utilize a biomimetic software liver. Results of virtual experiments provide compelling evidence that the prevailing explanation is insufficient. Without further studies in mice, we discovered a new, marginally more complex explanatory mechanism that met stringent tests of sufficiency. We argue that this virtual causal mechanism and the actual mechanism in mice are strongly analogous within and across multiple biological levels. Variants of the virtual mechanism provide possible explanations for the considerable variation in 24-hour necrosis scores among 37 genetically diverse mouse strains following a single toxic acetaminophen dose.

## Introduction

Acetaminophen (APAP)-induced liver injury (AILI) in mice is the widely used model for drug-induced liver injury in humans. There has been dramatic progress in identifying involvement of a variety of molecular level events and pathways [1–3]. To use that knowledge effectively in predicting injury and developing new treatment strategies, we need more credible explanations of how, when, and where key injury features emerge within hepatic lobules, and why humans exhibit a wide diversity of responses. A characteristic early feature is that necrosis, proceeded by covalent adduct formation, begins perivenous, adjacent to the central vein (CV) of hepatic lobules, and then progresses (radially) outward [4, 5]. Given that the fraction of APAP converted to the reactive metabolite (NAPQI: N-acetyl-p-benzoquinone imine) within hepatocytes increases from lobule’s periportal (PP) space to CV (called zonation), the prevailing mechanistic explanations assume that zonation of NAPQI formation is a necessary and sufficient prerequisite to explain early perivenous necrosis. That NAPQI zonation hypothesis provides an explanatory foundation for current efforts to better understand downstream complexities leading to tissue regeneration or liver failure. However, challenging that explanation directly in mice is currently impracticable because it requires exercising precise control over zonation of many mechanism features along with methods to measure intralobular features sequentially within the same mouse. Those barriers have impeded progress.

Here, we successfully circumvent those impediments using multi-attribute software-based experiments. We challenge the NAPQI zonation explanation by experimenting on a strongly analogous software mechanism (as distinct from being described mathematically), called NZ-Mechanism, that is instantiated within a virtual mouse. This software analog is engineered to be quantitatively and qualitatively biomimetic across all anatomical, hepatic zonation, and cell biology features currently believed relevant to challenging the NAPQI zonation hypothesis. Results from virtual experiments provide strong quantitative evidence that zonation of NAPQI formation alone is *insufficient* to explain early perivenous necrosis. A parsimoniously more complex explanatory mechanism is needed, one involving additional feature zonation. After testing several, we focused on two equally plausible mechanism zonation features, but they too failed to meet stringent tests of sufficiency. However, when the two zonation features that had failed were combined into a unified mechanism, sufficiency was achieved, thus demonstrating the potential power of the virtual experiment approach.

**Fig 1:**
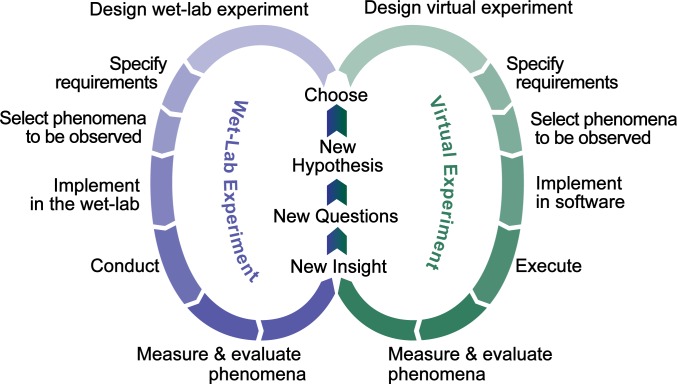
Cooperation and collaboration between wet-lab and virtual experiments in improving mechanistic explanations of phenomena. The workflow in each cycle has the same objective: challenge an explanatory hypothesis. Knowledge generated from right-side cycles is dependent on the combined strength of the four characteristics in Fig 3A. Knowledge gained from multiple right-side cycles can guide design of more efficient wet-lab experiments.

Physiologically reasonable perturbations of the merged mechanism caused significant changes in both the numbers of hepatocytes killed and the intralobular patterns of dead hepatocytes for a fixed toxic APAP dose of 300 mg/kg. Injury diversity was similar to that reported for humans [6, 7] and to differences in 24-hour necrosis scores among 37 genetically different mouse strains [7]. For the latter, we hypothesized that variation of lobule location-dependent features within the combined mechanism, resulting from changing selected mechanism configurations, can be sufficient to account for such necrosis score diversity. We present results of virtual experiments supporting that operating hypothesis with the intent that these results will help formulate new wet-lab experiments to further improve explanatory mechanism insight (Fig 1).

## Results

### Biomimetic NAPQI zonation hypothesis

**Fig 2:**
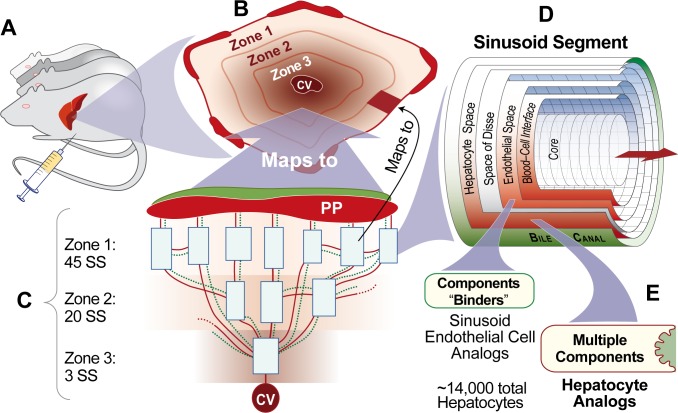
Mouse Analog components and their organization (A) Mouse Analog comprises a Liver, Mouse Body, as well as a space to contain dose; the space enables simulating intravenous, intraperitoneal, and intragastric dosing. During execution, each discrete time step maps to 1 second. (B) A Liver comprises Monte Carlo-determined Lobule variants. (C) A Lobule comprises a directed graph with a concrete Sinusoid Segment (SS) object (a software agent) at each graph node. The Lobular configurations used herein were validated earlier; they are the result of cycling many times through the Iterative Refinement Protocol (described later) and successfully achieving several quantitative Target Attributes having stringent Similarity Criteria [9–12]. All flow paths follow the directed graph. Bile (dotted green) flows separately from blood (solid red) but is not a factor for the hypotheses tested herein. Periportal (PP) to CV gradients provided intra-Lobular location information to each Hepatocyte. (D) Each Sinusoid Segment configures a parsimony-guided multilevel variety of components so that during execution it functions as an analog of sinusoid components and features averaged across many lobules; Sinusoid Segment dimensions are Monte Carlo determined to mimic hepatic variability. Cell objects occupy most of Endothelial Cell (99%) and Hepatocyte (90%) spaces. APAP objects enter and exit a Sinusoid Segment via Core and Interface, percolate stochastically through accessible spaces influenced by configuration-controlled local flow, and, if not metabolized, exit to CV and return to Mouse Body. (E) Cells in Endothelial space control APAP entry and exit and contain a probability-specified number of Binders; for this work, we only required that they bind and release APAP. Hepatocytes use three previously validated event management modules [13], which control 1) material entry and removal, 2) binding and object transformations, and 3) up- and down-regulation of events such as Metabolism (not used for this work).

**Fig 3:**
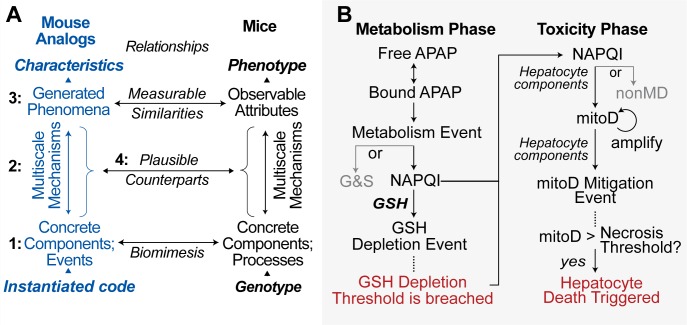
Intra-Hepatocyte events and Analog–mouse relationships (A) Experiments capable of challenging the NAPQI zonation hypothesis must demonstrate four characteristics. 1) Components are concrete and biomimetic. 2) Mechanism events during execution are observable and independent of phenomena being generated. 3) Qualitative and quantitative similarity (or lack thereof) can be established between target and Mouse Analog phenomena. 4) Means exist to incrementally strengthen claims that details of causal cascades in mice are strongly analogous [14]—quantitatively similar—to details of Mouse Analog’s causal cascade within and across multiple levels. (B) Virtual experiments designed to challenge the NAPQI zonation hypothesis focus on Metabolism Phase events and key early events within the Toxicity Phase of injury [1]. Although illustrated as a sequential cascade, each event executes independently in pseudo-random order each time step. All events are stochastic. Some event probabilities are Lobule location-dependent. An APAP object maps to a small fraction of an actual APAP dose, which for this work maps to 300 mg/kg. G&S objects represent APAP-glucuronide and APAP-sulfate plus all other inactive metabolites. A glutathione (GSH) Depletion event maps to depletion of a portion of a hepatocyte’s basal GSH. Mitochondrial Damage objects (mitoD) map to conflation of all influential damage products occurring within mitochondria [15]. Each mitoD may undergo one amplification event resulting in ≤ 6 additional mitoD; doing so enables downstream events to be finer grain than Metabolism Phase events. A mitoD Mitigation event maps to processes that advance recovery; it maps to an incremental reduction in mitochondrial disruption and damage.

Mouse Analog is the multiscale biomimetic software system (Fig 2) designed and built intentionally to be scientifically useful [8], especially, as in this work, for testing mechanism hypotheses of AILI, starting with the NAPQI zonation hypothesis: a configuration of NZ-Mechanism will be sufficient to explain early necrosis adjacent to CV. To first establish credibility that Mouse Analog can be used to challenge the NAPQI zonation hypothesis, we needed to achieve a variety of prespecified Target Attributes (performance requirements). A Target Attribute is a characteristic feature of APAP induced liver injury to which a prespecified Similarity Criterion is assigned. Each wet-lab measurement that we seek to mimic, such as hepatic extraction ratio, becomes a Target Attribute. The Similarity Criterion specifies the degree of similarity that must be achieved. An example is the mean virtual experiment measurement falling within ± 1 standard deviation of the mean wet-lab experiment measurement. The following are three of the Target Attributes achieved: measurements of APAP 1) hepatic extraction ratio, 2) intrinsic clearance, and 3) dose-dependent pharmacokinetic profiles (Supporting S1 Fig). By increasing the strength and variety of analogies between measurements made during experiments on Mouse Analog and corresponding measurements made during experiments on mice (Fig 3A), the credibility of explanatory inferences drawn from virtual experiments increases. Hereafter, Mouse Analog components and characteristics are capitalized to distinguish them from mouse counterparts; feature and parameter names are italicized.

For a particular Mechanism variant, Target Attributes are achieved by changing feature configuration values (Supporting S1 Table). Regardless of zonation, the probability of an event occurring within each Hepatocyte is fixed for the duration of the experiment. Probability of occurrence is the same within all Hepatocytes for events not subject to zonation. Note that to keep mechanisms parsimonious, some Fig 3B features, such as creation of Mitochondrial Damage (mitoD) objects, do not have direct mouse counterparts but instead subsume many.

### Zonation of Mechanisms

**Fig 4:**
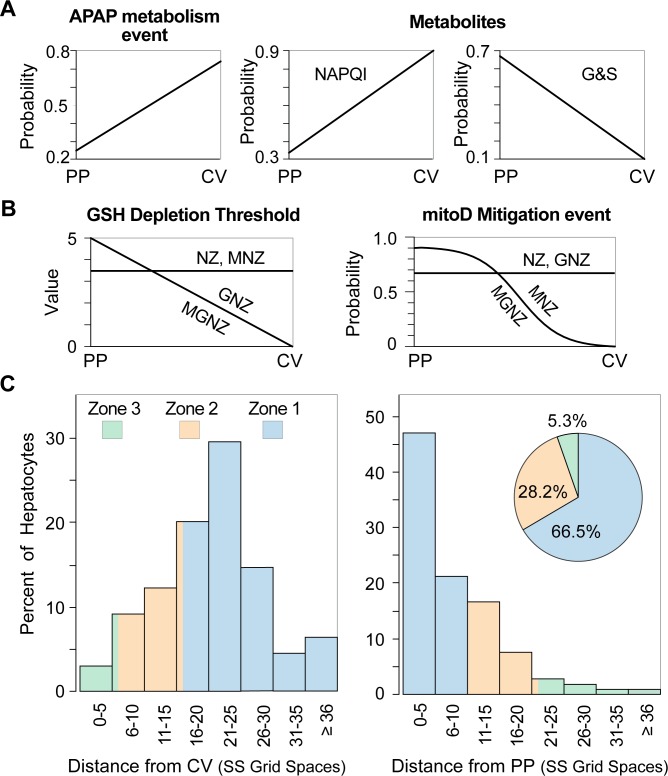
Location-dependent features (A) Only APAP metabolism configuration and the type of Metabolite formed are location-dependent in NZ-Mechanism. Target Phenomenon: pericentral necrosis begins first adjacent to CV and then moves (radially) outward in the PP direction. (B) GSH Depletion Threshold and probability of mitoD Mitigation event configurations are location-independent for NZ-Mechanism. However, one or both exhibit zonation (Methods) for GNZ-, MNZ-, and MGNZ-Mechanisms. (C) The relative locations of Hepatocytes are plotted in two different ways.

For NZ-Mechanism, the zonation of APAP Metabolism events (Fig 4A) causes the largest amount of NAPQI to be generated adjacent to CV. As depicted in Fig 3B, at each time step, each bound APAP may be Metabolized (maps to rate of metabolism); the probability of a Metabolic event is Lobule-location dependent, increasing from 0.35 to 0.95 PP to CV, which maps to increasing expression of participating metabolic enzymes. The probability that the Metabolite is NAPQI increases from 0.33 to 0.9 PP to CV (otherwise, G&S Metabolites are produced). That increasing probability maps to increasing expression of enzymes responsible for NAPQI formation, primarily CYP2E [1–4].

Glutathione (GSH) *Depletion Threshold* and probability of a mitoD Mitigation event are independent of Lobule location for NZ-Mechanism. Changing one or both alters total Hepatocyte Death but does not alter their Lobular locations. However, making one or both location-dependent (Fig 4B) changes the causal cascade and consequently alters Hepatocyte Death locations.

Because hepatic lobules approximate regular polyhedra with CV located centrally, the percent of a given lobule’s hepatocytes within different regions varies dramatically. The same is true within Mouse Analog Lobules (Fig 4C). Note that Lobule does not have a direct mouse counterpart (Methods). Rather, it maps to a small random sample of all hepatic periportal to perivenous flow paths. Because of periportal interconnections among sinusoids, the number of hepatocytes encountered by a compound such as sucrose moving through a lobule is typically greater than the number of hepatocytes along straight-through sinusoids. Flow through a Lobule enables APAP and other mobile objects to mimic that phenomenon.

### Testing four AILI Mechanisms

**Fig 5:**
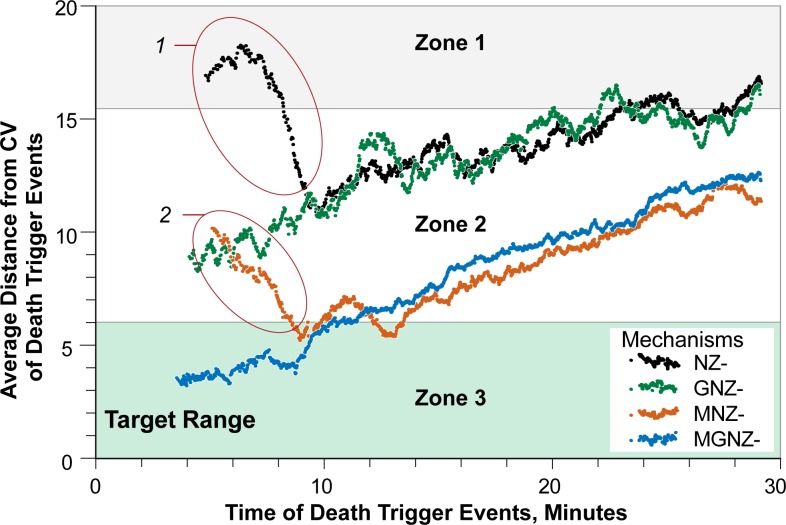
Three of four plausible Mechanisms falsified Shown are measurements from identical Mouse Analog experiments for which one of the four different Mechanism configurations was implemented. Average distances from CV (in Sinusoid Segment grid spaces) of Death trigger events each time step was recorded. Hepatocyte Death (plotted in Fig 6) becomes detectable *Death Delay* hours following its Death trigger event. Values shown are 100-second moving averages to reduce the considerable variability within and between simulation steps. Only MGNZ-Mechanism achieved the Target Phenomenon. Circled trends 1 and 2 help falsify NZ- and MNZ-Mechanisms.

The temporal patterns for average location of Death events and Death trigger events differ only by the value of *Death Delay* intervals, which range from 1.2 to 12 hours (see Death Delay subsection in Methods). We explain in the Death Delay subsection that measuring Death events while keeping measurement of events separate from the Mouse Analog mechanism is computationally expensive. Thus, we elected to use measurements of Death trigger events for Fig 5 to challenge the NAPQI zonation hypothesis. Even though the highest probability of NAPQI formation occurs adjacent to CV, NZ-Mechanism is falsified because early average trigger events failed to fall within the Zone 3 target range close to CV (Fig 5). Falsification is discussed further in the Validation subsection under Methods. Prior to about 6 minutes, the location of early average trigger events shifts toward CV, but at about 10 minutes the trend shifts in the PP direction, away from CV. Such an unusual pattern suggests that NZ-Mechanism may not be biomimetic. Seeking evidence that challenges that prediction would be problematic in mice at such early times. Nevertheless, what is the explanation for this Mouse Analog phenomenon?

Because of event stochasticity, some Hepatocytes at similar distances from the PP space will experience trigger events before their neighbors. Consider two regions: R_CV_ (nearer to CV) and R_PP_ (near PP space). The fraction of Hepatocytes experiencing an early trigger event will be larger in R_CV_. However, the total number of Hepatocytes in R_PP_ is larger (Fig 4C). Consequently, the number of early trigger events in R_CV_ and R_PP_ can be similar, sometimes even larger in R_PP_. Thus, the average location of all early trigger events (circled trend 1, Fig 5) can appear skewed toward R_PP_. Later, remaining R_PP_ Hepatocytes will be less vulnerable as the amount of APAP decreases, which causes the average trigger event location to shift toward CV. Concurrently, the fraction of Hepatocytes that have already experienced trigger events increased faster in R_CV_ than in R_PP_, explaining the direction change around 10 minutes.

The hypothesis that NZ-Mechanism alone is sufficient to explain early periportal necrosis is clearly falsified. We found no configurations that achieved that Target Phenomenon *and* shifted Hepatocyte Death patterns toward the CV (Fig 5). The results of these virtual experiments provide clear, strong evidence that an alternative, somewhat more complicated explanation is needed.

Having falsified NZ-Mechanism, we posited that at least *one additional* feature of the Mechanism must exhibit zonation. The following two (Fig 4B) seemed equally plausible. 1) GNZ-Mechanism specifies that GSH *Depletion Threshold* values decrease PP to CV *and* NAPQI formation values increase PP to CV. 2) MNZ-Mechanism specifies that each Hepatocyte’s ability to mitigate mitoD diminishes sigmoidally PP to CV *and* NAPQI formation increases PP to CV. Predecessors of MNZ-Mechanism employing a parsimonious linear mitoD Mitigation gradient failed to shift trigger events sufficiently in the CV direction. Using a sigmoidal distribution improved similarity to the Target Phenomenon, but not sufficiently. The results require that both MNZ- and GNZ-Mechanisms be falsified. We inferred that combining the two Mechanisms into MGNZ-Mechanism might be sufficient to achieve the Target Phenomenon, and identified values of MGNZ-Mechanism configurations that did so. Having achieved the Target Phenomenon, MGNZ-Mechanism stands as a plausible explanation of the target APAP-induced necrosis pattern.

### Explaining a plausible causal cascade

**Fig 6:**
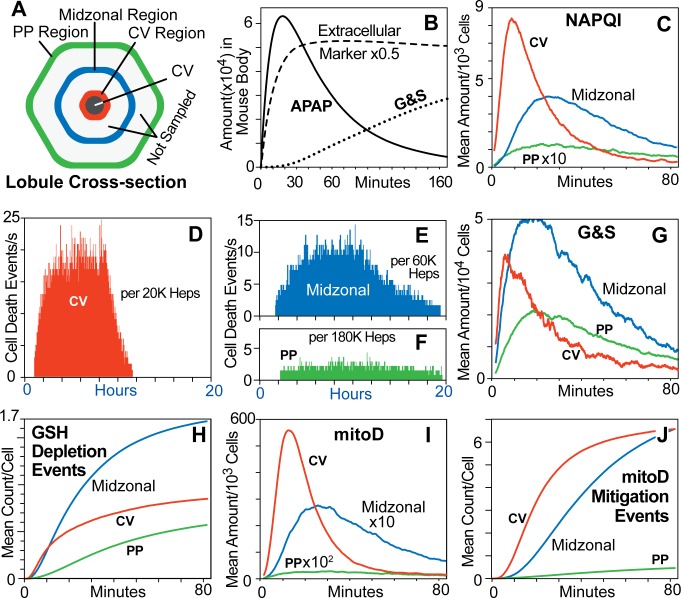
Cascading events within Lobules (A) During a Mouse Analog experiment that used MGNZ-Mechanism and started with a single toxic APAP dose (maps to approximately 300 mg/kg), measurements were made within the three illustrated 5-grid-space-wide regions: PP region adjacent to lobule entrance; CV region adjacent to CV; and Midzonal region in between. The experiment used 332 Monte Carlo variants of the same Mouse Analog. (B) Amounts in Mouse Body. The function of Extracellular Marker is analogous to that of an internal standard. It behaves the same as APAP, except that it is excluded from Cells and is not eliminated. The APAP profile during the experiment maps quantitatively to blood level profile in mice. G&S are transported out of Hepatocytes and accumulate in Mouse Body. Data in C and G-J are 100-second moving averages from the experiment described in A. (C) NAPQI profiles in each region reflect APAP profiles. APAP Blood levels adjacent to PP spaces are dramatically reduced as it distributes into the large number of accessible Hepatocytes. APAP in Blood in CV region partitions into far fewer Cells, so that per Cell concentration adjacent to CV at early times is actually greater than that in Cells adjacent to lobule entrance. (D-F) Histograms for number of Hepatocyte (Hep) Death events per second. Earliest Hepatocyte Deaths are seen at 1.2 hours after APAP dosing; Death trigger events occur earlier. (G) Amounts of G&S in each region. (H) Cumulative mean GSH Depletion events. (I) Mean amounts of mitoD: more than 5 mitoD per Hepatocyte triggers Death. Significant mitoD accumulation begins only after GSH Depletion. (J) Cumulative mitoD Mitigation events. Total Hepatocytes for 332 Monte Carlo variants: 4,648,000.

Contents of each Hepatocyte were measured within three 5-grid-space-wide bands (Fig 6A) during execution of MGNZ-Mechanism. The APAP pharmacokinetic profile (Fig 6B) is sufficiently similar to a plasma profile in mice [16] (Supporting S1 Fig). To mimic hepatic blood flow, a particular Mouse Analog feature configuration determines the fraction of APAP in Mouse Body that is transferred to Liver each time step. G&S are transported out of Hepatocytes and allowed to accumulate in Mouse Body. NAPQI peaking first in CV region (Fig 6C) is a consequence of differences in APAP exposure, feature zonation, and the fact that Hepatocytes that have experienced a Death trigger event stop Metabolizing APAP. Mean amounts of G&S per Hepatocyte (Fig 6E) also reflect NAPQI patterns. G&S amounts in CV region prior to 20 minutes are greater than PP region amounts, which may seem counter-intuitive. Zonation of Metabolism is one explanatory factor, but zonal differences in Hepatocyte numbers (Fig 4C) are more important: 742 in Zone 3, 3,948 in Zone 2, and 9,310 in Zone 1.

Prior to 80 minutes, Death has been triggered and APAP metabolism has been halted in most Hepatocytes in CV region but not Midzonal region. Thus, the value of cumulative Midzonal GSH Depletion events continues to increase after 80 minutes (Fig 6H). Having early mitoD values in CV region that are much larger (10x) than those in Midzonal region (Fig 6I) resulted in Hepatocyte Death occurring first near CV. The probability of a mitoD Mitigation event is smallest adjacent to CV, yet that is where the number of early Mitigation events is largest (Fig 6J). Therefore, although the probability of any one Mitigation Event is small, the cumulative number of such events is large.

### Inter-strain variability

**Fig 7:**
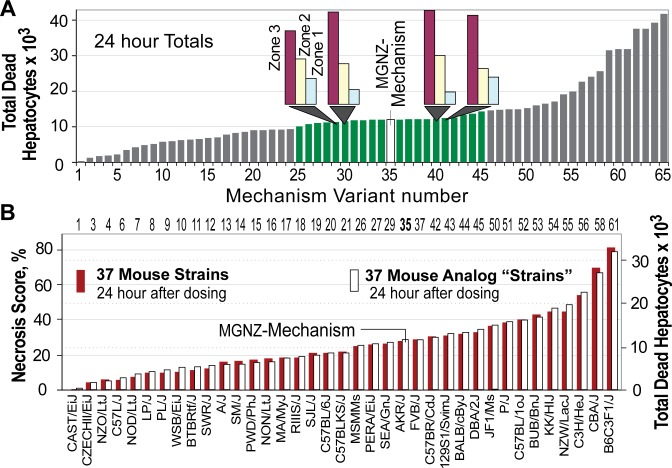
Mechanism diversity mimics diversity of toxicity among genetically different mouse strains (A) Total Dead Hepatocytes (out of a total of 168,000) are plotted 24 hours after simulated oral APAP dosing using 64 plausibly biomimetic variants of MGNZ-Mechanism. Examples of the configuration used are shown in Supporting S3 Fig. Each variant represents a virtual mouse strain. Based on reported variability between mice in the same experiment, we specified that mean toxicity measurements for any variant that was within 20% (a judgment call) of mean values from MGNZ experiments (shaded green) could be determined experimentally indistinguishable. Inserts: Mechanistic consequences of changes to configurations are brought into focus by comparing Deaths per Zone rather than totals. (B) Shown are mean necrosis scores (left axis; n = 3–4/strain) data from Harrill et al. [7] along with one of the 64 Mouse Analog variants from A selected as described in the text. Their identifying Mechanism variant numbers are listed at the top.

The space of MGNZ-Mechanism configurations contains both biomimetic and non-biomimetic variants. A variant that, for example, eliminates zonation of APAP metabolism (Fig 4A) is non- biomimetic. To test sensitivity and robustness of the MGNZ-Mechanism configuration, we conducted experiments using 64 plausibly biomimetic Mouse Analog variants of the MGNZ-Mechanism configuration in which values assigned to one-to-seven of ten influential configurations (shown in Supporting S2 Table) were changed. Each variant represents an arbitrary virtual mouse strain. Most changes included adjusting one or more zonation configuration. The Mechanism configurations included the five in Fig 4A and 4B plus the following: the probability that a NAPQI object will react, *Death Delay* value, the probability that a NAPQI reaction product will be mitoD or nonMD (non-mitochondrial damage products, discussed below), the probability of a nonMD Mitigation event, and/or *Death Trigger Threshold* value. Total Dead Hepatocytes 24 hours after dosing for all 64 variants were ordered in ascending order and plotted (Fig 7A). Comparing Deaths per zone rather than total Deaths (inserts, Fig 7A) provides concrete evidence of differences in how events within each Mechanism variant unfolded. Supporting S2 Fig provides additional examples.

We compared those results to data from Harrill et al. [7] (red bars, Fig 7B) in which mean necrosis scores of 37 different mouse strains were recorded 24 hour after dosing with 300 mg/kg of APAP administered intragastrically. We assumed a direct correlation between necrosis score and both numbers of necrotic cells (in mice) as well as total Dead Hepatocytes (Fig 7B, right axis). All Mouse Analog data in Fig 7A were scaled the same so that at least one (of the 64) Mouse Analog bar heights was similar to the smallest and largest values in Fig 7B. We arbitrarily matched the 24-hour necrosis score for strain FVB/J to total Dead Hepatocytes to Mechanism variant #37, and then selected the one variant that provided the closest match to the necrosis score for each of the other mouse strains (white bar).

## Discussion

Our intent is that parallel virtual and wet-lab experimentation will integrate into a faster and more effective scientific approach [17] (Fig 1). Most researchers seem to prefer including ample realistic detail in explanatory models. However, mechanisms like those employed in Mouse Analogs are scientifically preferable and more informative because they are only as detailed as necessary for explaining a particular phenomenon or challenging a particular hypothesis. A validated model Mechanism with limited detail subsumes many different yet equally plausible more detailed variants. When we falsify such a Mechanism (show that it is inadequate for its Target Attributes), we significantly shrink the space of equally plausible variants by eliminating whole classes of more detailed variants.

The results in Fig 5 document the first use of virtual experiments to challenge competing biomimetic Mechanism hypotheses. The fact that three were falsified demonstrates how virtual experiments can shrink the space of plausible explanatory Mechanisms and facilitate the more fundamental work of wet-lab experimentation.

When we started, we expected Mouse Analogs using NZ-Mechanism to show Hepatocyte Deaths occurring first near CV. We acquired new knowledge by examining why it, as well as MNZ- and GNZ-Mechanisms, failed to mimic that phenomenon. Explanatory insight was further improved by examining *how* MGNZ-Mechanism successfully achieved the Target Phenomenon. An example is the importance of zonated repair pathways in mitigating damage (Fig 6). We anticipate that other comparably parsimonious yet somewhat different Mouse Analog Mechanisms can do the same.

The Iterative Refinement Protocol (described under Materials and Methods) outlines how to identify and challenge comparably parsimonious explanations of target phenomena. Mouse Analog concreteness, coupled with the ability to observe and measure propagating causal events, makes it easy for experts and non-experts to form and discuss opinions about the credibility of similarities between virtual experiment results and those from real or envisioned wet-lab counterparts.

NZ-Mechanism was falsified because a small fraction of Hepatocytes in Zones 1 and 2 experienced an early Death trigger event (Fig 5) and consequently will experience an early Death event. The same phenomenon may occur in mice, but it has escaped detection because experiments were focused elsewhere. If deemed important, that phenomenon—that prediction—could be challenged directly using wet-lab experiments.

During execution, MGNZ-Mechanism is a concrete hypothesis for how key features of APAP hepatotoxicity in mice are generated (Fig 3A). Suppose that strong analogies *do* exist between virtual and real mice. It is clear from concurrent differences within the three regions (Fig 6C–6H) that explanatory inferences drawn from whole liver measurements or hepatic biopsies will at best be flawed and likely misleading.

Differences in zonal predictions can guide design of wet-lab experiments intended to challenge and possibly falsify the MGNZ-Mechanism hypothesis. For example, 1) at 30 min post-dose, GSH Depletion in the Midzonal region is significantly greater than in either CV or PP regions; and 2) at 10 min post-dose, counts of mitoD objects (maps to mitochondrial damage) in CV Hepatocytes are an order of magnitude greater than in PP Hepatocytes. Such experiments will be win-win: no matter the outcome, we will have useful new knowledge, and the new results are expected to motivate additional rounds of virtual experiments [17] (Fig 1). Note that, absent the data in Fig 6 there would be no basis to undertake such narrowly focused experiments.

Within individuals, expressions of hepatic proteins are known to change significantly in response to a variety of environmental and health factors. We simulated possible examples (Fig 7A). Genetic differences among mouse strains that influence hepatic zonation more than they influence particular pathways or molecular level events may account for the diversity of differences in necrosis scores (Fig 7B). However, considerable uncertainty remains. As illustrated by the green bars in Fig 7A, for each Mouse strain explanation, there will exist a sizable set of Mechanism variants that would be equally explanatory. Further, MGNZ-Mechanism is just one realization drawn from a plausible yet finite Mechanism space. Automated methods, as they become available, can be used to more systematically explore that space, prior to or in parallel with new wet-lab experiments to challenge MGNZ-Mechanism.

The data in Fig 7 demonstrate that, when targeting just one attribute, such as total Hepatocyte Death 24 hours post-dose, modest configuration changes for two or more Mouse Analog features can have counterbalancing consequences resulting in altered event details yet essentially no significant change in the measured feature. On the other hand, modest changes can have synergistic consequences resulting in a 3-fold change or more. Toxicity enhancing synergistic changes in cascade zonation, possibly initiated by changes in environmental and health factors, may be a contributor to idiosyncratic drug induced liver injury. In combination with the adaptation hypothesis [18], such change may help explain how and why only a small percentage of susceptible individuals develop overt liver injury.

## Materials and Methods

### Requirements

Methods stem from four requirements [19], which are needed to implement the use cases illustrated in Fig 3B. Meeting them is outside the scope of systems biology models currently in use to predict APAP hepatotoxicity [20, 21]. We use the virtual experiment approach described by Kirschner et al. [17] along with the enhanced strategies detailed by Petersen et al. [22, 23]. Feature names (Supporting S1 Table) are italicized.

1. Darden argues that a biologically explanatory mechanism will exhibit the fourteen features listed in Supporting S3 Table [24]. NZ-Mechanism must exhibit all of those features. Examples are listed in Supporting S3 Table.
2. Components and spaces (Fig 2) are concrete, biomimetic [25, 26], and sufficiently modular to facilitate analogical reasoning [14, 27]. These characteristics allow:
  · Defining and annotating mappings (Fig 3)
  · Reusing components to represent counterparts in different species
  · Challenging competing hypotheses through experimentation (Fig 1)
  · Representing different experimental designs and protocols
  · Changing Mechanism detail (granularity, resolution)
  · Exploring new intervention scenarios
  · Testing (verification) of components in isolation as well as within a configured Mouse Analog
  · Versioning, in which one component can evolve independent of others
  · Comparing and contrasting predictions
  · Increasing the strength of the explanatory and predictive analogies in Fig 3A over time
3. Phenomena measured at a higher level of organization arise mostly from local component interactions at a lower level of organization.
4. Different objects mapping to different chemical entities can be used simultaneously. Quasi-autonomous components (i.e. agents such as Hepatocytes) recognize mobile objects and appropriately adjust their response.

To achieve Requirement 2, Mouse Analogs are written in Java, utilizing the MASON multi-agent simulation toolkit [28]. In silico experiments are run using virtual machines [29] on Google Compute Engine, running 64-bit Debian 7. For longer simulations (e.g., Fig 6) Monte Carlo trials are run in parallel. The data presented herein along with Mouse Analog code are available [30].

### Iterative Refinement Protocol

We started with a previously validated version of Liver [9]. The goal of an Iterative Refinement Protocol cycle is to refine a formulated explanatory Mechanism by achieving Target Attributes, thus completing one right-side cycle in Fig 1. A concrete software mechanism can be falsified—shown inadequate for its Target Attributes—in two ways: 1) it cannot exhibit a Target Attribute and/or 2) we fail to discover a Mouse Analog configuration that achieves all Target Attributes.

Before starting, specify Target Attributes to be explained and rank-order them in terms of expected difficulty to achieve. For each Target Attribute, specify initial and possible subsequent Similarity Criteria.

1. Falsify the previously validated Analog by either 1) increasing Similarity Criterion stringency or 2) adding a new Target Attribute. Each falsification improves credibility incrementally and shrinks the space of plausible, explanatory Mechanisms.
2. Specify a revised explanatory Mechanism; e.g., if we do *XYZ*, then the altered Mouse Analog will achieve the Target Attribute (along with previously achieved Target Attributes).
3. Outline a revision plan, which may include revising modules, components, use case(s), feature configurations, etc. Implement the revised Mechanism. Adhere to a strong parsimony guideline; so doing helps separate causes from effects. Take small steps that mostly fail. Making the updated Mechanism too fine grain expands the space of plausible configurations beyond one’s ability to manage efficiently. It is from encountering and overcoming iterative falsifications that explanatory insight into both Mouse Analog and AILI improves.
4. Based on the current objective and available measurements, reformulate Similarity Criteria; e.g., measurements fall within ± 1 standard deviation of the Target Attribute. For time-course data, specify a percentage (e.g., 80%) of Analog measurements that must fall within the prespecified range. When first applying a quantitative Similarity Criterion, make it weak. At Step 6, stringency can be increased for the next cycle.
5. Conduct and evaluate many simulations—virtual experiments.
6. When the revision fails, return to Step 2. A failed revision provides new knowledge and shrinks plausible Mechanism space. When successful, we have achieved a degree of validation and incrementally improved analogical credibility. Return to Step 1.

### Liver and lobular form and function

Liver composition (Fig 2) is now stable and robust, having already achieved several Target Attributes [9–12, 31]. Bile Network does not influence the four Mechanisms. Because rat and mouse lobule structure and organization are similar, Liver can be used in simulations of phenomena measured in rats and mice. By altering analog-to-referent mapping functions [25], we can change experimental context. A Lobule maps to a small random sample of all lobular flow paths. The 3-zone network has 68 nodes, 45, 20, and 3 in Zones 1, 2, and 3, respectively. Nodes are connected using 99 edges: 55 from Zone 1-to-Zone 2; 10 within Zone 1; 24 from Zone 2-to-Zone 3; 10 within Zone 2; and 0 within Zone 3. To represent both uncertainty and variability, edges between Sinusoid Segments are randomly assigned at the start of each Monte Carlo execution. Having edges and Sinusoid Segment sizes assigned randomly at the start of each experiment simulates lobular variability within and between livers. See *Validation* subsection below for additional detail.

### APAP metabolism

Hepatocyte objects, excluding Fig 3B capabilities, are identical to those described in Petersen et al. [13, 22]. Hepatocytes contain four physiomimetic modules [13]: InductionHandler, EliminationHandler, MetabolismHandler, and BindingHandler. The events and features illustrated in Fig 3B were added incrementally.

Liver sinusoidal endothelial cells have been implicated in AILI. They contain CYP2E1 and GSH, although the relative amounts are very small compared to hepatocytes. An unanswered question is whether lobular differences in CYP2E1 and GSH within liver sinusoidal endothelial cells contribute to zonation of injury [32]. Addressing that question is feasible using Mouse Analog, given Target Attributes specific to liver sinusoidal endothelial cells, but it is outside the scope of this work. Adhering to a strong parsimony guideline, and given that the vast majority APAP metabolism occurs within hepatocytes, we specified that all relevant APAP metabolism and GSH Depletion occur within Hepatocytes. Within Mouse Analog Endothelial Cells, we specified that only nonspecific APAP binding occur. It is straightforward to add modules to Endothelial Cells and configure them appropriately should evidence become available that falsifies those specifications.

The first performance goal was to discover Metabolism phase (Fig 3B) feature configurations that, upon achieving Target Attributes, would remain unchanged during experiments to challenge Mechanisms intended to explain necrosis patterns. There is quantitative variability (within and between experiments) in zonal measurements of CYP2E1 (primarily responsible for formation of NAPQI) [33], the relative proportion of the three primary metabolites (glucuronide, sulfate, and NAPQI) [34], hepatic clearance, and hepatic extraction ratio. However, there is qualitative agreement on their trends.

An APAP object maps to a tiny fraction of the 300 mg/kg dose. There is a direct mapping between the probability of an unbound APAP object being metabolized each time step (1 second) and amounts of metabolic enzymes [13, 22]. In mice, CYP2E1 levels per hepatocyte increase PP to CV by 2 to >10 fold. We specified this Target Attribute: probability of an APAP metabolic event generating NAPQI increases at least three-fold PP to CV. All other metabolites are lumped together and called G&S (maps mostly to the glucuronide and sulfate metabolites). In most reports, inactive metabolites are estimated to account for up to 85% of a dose, with NAPQI accounting for the balance. We specified that total NAPQI, as fraction of dose, be within 0.15–0.4; a large range was needed to ensure sufficient numbers of NAPQI are generated when dose is reduced. A Target Attribute is that toxicity be reduced by about 50% when the APAP dose is reduced by 50%.

This Target Attribute was the most demanding: APAP hepatic extraction ratio = 0.6 ± 0.06. To simplify achieving that Target Attribute, we used a virtual single pass Liver perfusion protocol with a constant rate of APAP input. When APAP outflow stabilized within 34–46% of input values, the Target Attribute was reached. For convenience, we began the initial Iterative Refinement Protocol cycle by specifying that APAP Hepatic Clearance be similar to that of prazosin, for which the Liver had already been validated [10–12]. We cycled through the Iterative Refinement Protocol seeking changes to configurations that would enable achieving additional Target Attributes specified below under Mouse Body. The resulting configuration values are illustrated in Fig 4A and 4B.

### Zonation and GSH Depletion

Lobules have a biomimetic PP to CV gradient [13] (Supporting S4 Fig), which Hepatocytes can use to create feature zonation. It maps to measures of one or more common blood attributes, such as pO_2_ [35]. However, because we needed the flexibility to explore multiple plausible, feature-specific PP to CV gradients for several different events, those in Fig 4A and 4B were implemented explicitly as functions of path length from analog Lobule PP entrance to the current Hepatocyte’s position. Path length (distance from PP entrance in grid spaces, *dPP*) is specified by this Hepatocyte’s distance from the inlet of its Sinusoid Segment (SS) plus the averages of the lengths of all Sinusoidal Segments in each of the previous zones. I.e., *dPP*(SS^z^) = $\sum_{i=z}^{1}{\left\langle \left|{SS}_{j}^{i-1}\right|\right\rangle }_{j}$, where SS^z^ is a given Sinusoid Segment in Zone z and |SS| is its length and *j* indicates each SS in zone *i*; *j* indicates each SS in zone *i*; and *dPP*(H^ss^) = x^ss^ + *dPP*(SS^z^), where H^ss^ is a Hepatocyte in a given Sinusoid Segment, and x^ss^ < |SS| is a position along the length of a Sinusoid Segment. The angle brackets indicate the average of its contents. Each gradient has a shape (linear or sigmoidal) and its own start and end values, which are then scaled over the common path length computation. Gradients are turned on or off independently by specifying their start and end values. Once these feature-specific gradients have stabilized, they can be replaced by rules that use the common biomimetic gradient.

We started with a simple parsimonious operating hypothesis: when specific damage products accumulate within a Liver above some threshold, Death will be triggered irreversibly. The evidence shows that accumulation of APAP-induced damage products in mice occurs after available GSH has been sufficiently depleted. By analogy, we implemented a new feature: at each time step, with *p =* 0.5 (specified arbitrarily because we had no wet-lab data to guide doing otherwise), each NAPQI object may be destroyed, which maps to in vivo depletion of a fraction of hepatocyte’s available GSH. A NAPQI destruction event is also a GSH depletion event. A small GSH *Depletion Threshold* value maps to mice that are very sensitive to APAP hepatotoxicity; a larger GSH *Depletion Threshold* maps to increased resistance. The *Threshold* value needed to be small enough to allow sufficient accumulation of Damage products (below), but large enough to achieve this Target Attribute: toxicity is reduced by about 50% when the APAP dose is reduced by 50%. Setting GSH *Depletion Threshold* = 5 for NZ-Mechanisms proved adequate. In wet-lab studies, GSH is often measured in whole liver homogenates. To make NZ- and MNZ-Mechanisms more directly comparable to GNZ- and MGNZ-Mechanisms at the level of whole Lobule measurements, we specified that *Depletion Threshold* = 3.5 for NZ- and MNZ-Mechanisms, which is the average value for all Hepatocytes using GNZ- and MGNZ-Mechanisms. For some cases in Fig 7A, GSH *Depletion Threshold* values at the Lobule’s entrance of 8 or 3 are used (Supporting S2 Table).

### Damage products trigger Hepatocyte Death

Each time step after *Depletion Threshold* is reached, with *probability to react =* 0.5, each NAPQI object may be destroyed and replaced by a Damage product. Evidence implicates necrosis being triggered by mitochondrial damage. We parsimoniously specified that there be two classes of Damage product: “mitochondrial damage products,” called mitoD, and “non-mitochondrial damage products,” called nonMD. When a NAPQI object is destroyed, it is replaced with either a nonMD or mitoD object selected randomly (*probability* = 0.5); we had no wet-lab data to guide specifying differently.

To trigger Death, we implemented an analog counterpart to this simple yet widely accepted mechanism: upon accumulation of sufficient mitochondrial damage, a tipping point is reached and necrosis is triggered irreversibly. Triggering necrosis within one mouse hepatocyte requires reaction of possibly hundreds (or more) of NAPQI molecules, which is a very tiny fraction, *f*, of the administered APAP. One analog NAPQI object maps to a very small fraction, *f*_A_, of that same APAP dose. For mice resistant to APAP hepatotoxicity (case 1), it is possible that *f* > *f*_A_. When referent mice are much more sensitive to APAP hepatotoxicity (case 2), it is likely that *f*_A_ > *f*. For case 2, replacing one NAPQI with one mitoD will be more than enough to trigger a Death event. Thus, the resolution [17] of one NAPQI → one mitoD is inadequate to simulate toxicity phase events. We solved that problem by specifying that each mitoD be amplified: one NAPQI → (1 + *n*) mitoD. Specifying that *n* be a random draw from either a uniform (1, 6) distribution enabled achieving Target Attributes. The mitoD amplification step is an example of making an analog Mechanism locally finer grain (increasing resolution) without changing granularity elsewhere.

As with *Depletion Threshold*, the *Death Trigger Threshold* value needed to be small enough to allow sufficient accumulation of mitoD without a trigger event, but also large enough so that toxicity is reduced by about 50% when APAP dose is reduced by 50%. Setting *Death Trigger Threshold* = 6 proved adequate. For some cases in Fig 7A, larger *Death Trigger Threshold* values are used.

### Damage Mitigation events

Hepatocytes utilize multiple mechanisms to mitigate or reverse different types of damage, ranging from up-regulation of GSH synthesis to mitochondrial autophagy [36]. Consistent with our strong parsimony guideline, we started with a single mitigation Mechanism, which maps to a conflation of all actual mitigation/recovery mechanisms. Each time step, with *p =* 0.5 (specified arbitrarily), each nonMD and mitoD object may be destroyed. For NZ-Mechanism, lacking wet-lab data for guidance, we made *probability-of-Mitigation* event for each nonMD and mitoD at each time step independent of Lobule location.

Because hepatocytes closer to CV are known to experience increasing oxidative stress, we inferred that mitigation mechanisms would be more robust closer to CV. However, when we increased *probability-of-mitoD-Mitigation* event (simply *p-mitoD-Mitigation* below) PP to CV, the locations of Dead Hepatocytes were shifted further in the PP direction, relative to the NZ-Mechanism data in Fig 5. To shift Death trigger events in the CV direction, it was necessary to decrease *p-mitoD-Mitigation* PP to CV. Instantiating a linear 0.9-to-0 decrease in *p-mitoD-Mitigation* failed to produce a sufficient shift. We considered several options for moving mean trigger events toward CV. E.g., keep *p-mitoD-Mitigation* constant at 0.9 through Zone 2 and then decrease it linearly to 0 at CV. We rejected that feature because, absent any supporting observation, the risk seemed too great that it would be non-biomimetic. We considered adding a new biomimetic feature or event, but doing so conflicted with our strong parsimony guideline. Following several exploratory Iterative Refinement Protocol cycles, we opted for specifying that *p-mitoD-Mitigation* for MNZ- and MGNZ-Mechanisms decreases PP to CV following a reverse sigmoid with the inflection centered approximately midway in Zone 2. However, there is neither supportive nor refuting evidence for that configuration; therefore, this explanatory Mechanism hypothesis needs to be challenged. We could posit that the efficiency of one or more processes for mitigating normal mitochondrial dysfunction decreases PP to CV [36, 37] and that one or more of the processes that maintain necrosis-triggering pathways is preferentially sensitive to NAPQI damage. Consequently, their combined effect maps to *p-mitoD-Mitigation* decreasing sigmoidally PP to CV in Mouse Analog.

As with the GSH *Depletion Threshold*, to make NZ- and GNZ-Mechanisms directly comparable to MNZ- and MGNZ-Mechanisms at the whole Lobule level, we determined that the average *p* value for all Hepatocytes using is 0.67. Therefore, we specified that *p-mitoD-Mitigation* be 0.67 for NZ- and MNZ-Mechanisms in Fig 5 rather than 0.5.

### Mouse Body

Connecting Liver to Mouse Body produces Mouse Analog. Mouse Body contains a space that maps to all extrahepatic tissues including blood along with a space to contain dose, which enables simulating intravenous, intraperitoneal, and intragastric dosing. When additional details are required, new objects can be added without influencing preexisting Liver Mechanisms. To achieve APAP pharmacokinetic attributes, we adjusted values of two configuration features: one controls Absorption into Mouse Body and the other controls metering of APAP from Mouse Body to the Lobule’s entrance. The latter maps to hepatic blood flow. We specified that Absorption be first order and rapid.

We prespecified two Target Attributes: 1) mimic single dose APAP pharmacokinetics in mice [16] and 2) observe characteristic dose-dependent pharmacokinetics for APAP in Mouse Body. For (1), we prespecified that Mouse Body measurements be within one standard deviation of mean wet-lab values. Supporting S1A Fig shows that the first Target Attribute was achieved. For (2), we found no fine grain dose-dependent pharmacokinetic data in mice to use as direct validation targets. However, there is pharmacokinetic data in rats [38, 39] demonstrating characteristic dose-dependent pharmacokinetics. We reasoned that, by using a simple mouse-to-rat scaling, reported dose-dependent pharmacokinetic data in rats [38, 39] could serve as alternative targets, despite the fact that the metabolism and toxicity of APAP in mice and rats are quite different (we are targeting dose-dependent APAP clearance, not metabolite types). For comparable mg/kg doses, the plasma half-life of APAP in rats is approximately 3x-5x that in mice [40]. The dose of APAP objects used in Fig 6B maps to 300 mg/kg in mice. Using the median value of 4x, we determined that dose should scale to approximately 75 mg/kg in rats. Twice that dose of APAP objects should map to approximately 150 mg/kg, and half that dose should map to approximately 37.5 mg/kg in rats. Supporting S1B and S1C Fig show that characteristic dose-dependent pharmacokinetics is observed. Attaining Target Attributes from different species increases credibility and documents achieving the second capability under Requirement 2.

### Death Delay

There are qualitative but not quantitative time-course data for relative amounts of necrosis in mice. Necrosis is not detectable during the first hour following a toxic but non-lethal APAP dose of 300 mg/kg, and there is no evidence of significant further induction of necrosis 12 hours after dosing [3]. Covalent adduct formation and histological evidence of hepatocyte damage always precedes measurable necrosis by tens of minutes to a few hours. That time interval maps to *Death Delay*. To specify the *Death Delay* feature, we needed a Target Attribute estimate for percent total necrosis as a function of time post-dose. Target range estimates (see Supporting S5 Fig) were provided by coauthor Kaplowitz for percent total necrosis at several times post-dose. The prespecified Similarity Criterion was that cumulative mean Death events fall within the estimated Target Attribute range. That Target Attribute was achieved in two different ways (Supporting S5 Fig): draw each Hepatocyte specific *Death Delay* value from a uniform (a pseudo-random draw from uniform [1.2, 12] hours) or a normal distribution. Although the second seemed more realistic, we used the first because it is more parsimonious and doing so enabled achieving the Target Attribute range.

Necrosis is the target wet-lab phenomenon. Our working hypothesis is that necrosis is a *process* that is triggered by early damage and that the wet-lab measurement is detecting a late stage in that process. *Death Delay* maps to the *process* of necrosis. The time of an Hepatocyte Death event = time of that Hepatocyte’s Death trigger event + a pseudo-random draw from [1.2, 12]. In Fig 5, we elected to focus on Death trigger events rather than time of an Hepatocyte Death events for three reasons. 1) Although there is no current wet-lab method to measure its short transient duration, the trigger event is a key causal event. 2) We know that final Lobular locations of trigger events and Dead Hepatocytes are the same. 3) To make the virtual experiment as much like a wet-lab counterpart as possible, we strive to measure the analog in the same way one measures the referent. Conventional models are not *measured*; rather, they simply output variables (e.g. concentrations of species) each time step. In contrast, Mouse Analog is *measured*. Doing so during executions requires keeping Death event measurements separate from Analog Mouse Mechanisms. Measuring Death events requires a high frequency polling effort, where during one poll the observer agent records a Hepatocyte in a particular location as not Dead, and the next poll records that Hepatocyte as Dead. Such polling increases considerably the time required to complete each simulation experiment.

### Data types, reuse, code availability, and sharing

Mouse Analogs are treated as a form of data, using both the implicit schema of Java, JavaScript, and R and the explicit schema of the configurations. Mouse Analogs and configuration data are maintained, archived, and released using the Subversion version control tool in two repositories, one private (Assembla) for rapid and prototyping development with project partners and another public for collaboration [40, 41]. Input-output (I/O) data is handled separately. Smaller data sets are stored in simple CSV. I/O data is tightly coupled to experiments and configuration details, requiring a common versioning system, aggregating all three data types. Versioned I/O data is archived as downloadable packages.

The entire toolchain, including the operating system, used for Mouse Analogs, configurations, and I/O handling is open-source, thereby ensuring repeatability. Similarly, all project generated and released data is available to be licensed as open data. Mouse Analogs are built for, maintained, and executed in a cloud environment (e.g., Google Compute Engine) to ensure platform and infrastructure repeatability across experiments, project team members, partners, and the wider community.

### Quality assurance and control

Regression and unit tests are special cases of canonical use cases (e.g., a single pass Liver perfusion experiment). While use cases are instances of the class of experiments to which Mouse Analogs are being applied (e.g., AILI), they also provide the measures by which the software and methods are maintained. Each toolchain iteration can execute the canonical use cases so that current results can be compared to prior results for which some degree of credibility has been documented. Significant variations are documented, investigated, and explained. While the majority of variations are the result of model iterations, the process does catch artifacts introduced by changes.

Unexpected variations in results during an Iterative Refinement Protocol cycle first trigger a software verification process designed to test the toolchain from the most general and widely used, up to the most specific tool: machine, OS, compiler, libraries, simulator, and model. Absent unexpected variations, that same process is applied at least yearly. So doing is rigorous and avoids wasteful iterations that result from assuming that all variations are caused by the most specific layer. Should anomalies occur at the Mouse Analog layer, they would trigger a source, data, configuration setting, and trace review in comparison with the design of experiments. Any unexpected variations that survive the verification process are then submitted to an Iterative Refinement Protocol based falsification battery designed to invalidate the Mouse Analog as compared to wet-lab observations. Results satisfying canonical use cases, but yielding unexpected variation as a result of changes to Mouse Analog or toolchain, provide material that can be used to formulate a useful hypothesis.

### Building Mouse Analog credibility

#### Prediction

A prediction is an analog system behavior that is tested in the future. Each Mouse Analog execution generates predictions. However, Requirements make clear that Mouse Analogs are primarily exploratory and explanatory devices. Thus, the focus in this work is more on improving explanatory Mechanisms for how AILI features may be generated. When needed or justified, it is easy to add details that improve apparent realism (e.g., see Pogson et al. [26]). However, we strive to keep analog Mechanisms parsimonious in order to retain scientific usefulness. As explanatory credibility increases, additional effort can be invested in achieving a more precise prediction of hepatotoxicity features.

#### Validation

Mouse Analogs are both structural and behavioral hypotheses for mice (Fig 3). As such, validation methods (success at Step 6 of the Iterative Refinement Protocol) include both explanatory and predictive types, and are both qualitative and quantitative. Achieving Target Attributes is the objective of each Iterative Refinement Protocol cycle. But iterative refinement is an ongoing process. To further improve explanatory insight, we must challenge Mouse Analog. Recall that Mouse Analog is an instantiated hypothesis. If it fails the challenge, then some aspect of the instantiated hypothesis is false. We use a new Iterative Refinement Protocol cycle to overcome that failure. Thus, in advancing the science, validation is just one part of an ongoing two-part process [8]. Falsification is the second essential part. At Step 1 of the Iterative Refinement Protocol, we falsify the Mechanism that was validated previously at Step 6 by either adding a new Target Attribute or increasing the stringency of a Similarity Criterion. During any iteration of the Iterative Refinement Protocol, the Mouse Analog Mechanisms currently in use are those that have survived prior testing. The number and type of tests and/or falsification challenges a Mouse Analog has survived will establish its credibility. Making these validation-falsification cycles explicit, even when seemingly minor, improves credibility by providing a workflow record that ensures that the work can be reproduced. It also provides a record of the engineering and biological reasoning used.

Quantitative tests consist of well-defined Similarity Criteria comparing Analog output with wet-lab data, such as that described and cited herein. Each Mouse Analog carries an inscribed variability from probabilistic configurations and Monte Carlo sampling. Hence, surviving a quantitative test against wet-lab data requires two Target Attributes for each pairing between an analog use case (e.g. mimic a wet-lab experiment) and the wet-lab dataset (measurements of the particular Target Phenomenon):

Target Attribute 1: The actual similarity being measured as a sample-space dependent, well-defined distance between measurements of a wet-lab phenomenon and measurements of Mouse Analog’s phenomenon; and
Target Attribute 2: The extent to which Mouse Analog and wet-lab variations are comparable with the corresponding uncertainty.

Variation can be intra- and inter-individual, or within and across experiments as in Fig 7. Where possible, as demonstrated by Petersen et al. [13], components are alternately composed to achieve in vitro and in vivo Target Attributes, thereby testing across two different use cases. So doing provides an aspect-oriented validation method that, though behavior-based for each use case, facilitates multi-scale and structural validation (both qualitative and quantitative), and translation to application.

Qualitative tests consist of characteristic features of the wet-lab observations that can be generalized to a variety of wet-lab scenarios. Examples applied to the 4 Mechanisms (NZ, MNZ, GNZ, MGNZ) include requiring that APAP metabolism and NAPQI production increases PP to CV and necrosis occurring first close to CV.

#### Verification

Using pseudo-random generators facilitates verification. Because Mouse Analogs constitute concrete structural Mechanism hypotheses, verification is limited mostly to mathematically well-defined sub-component requirements. Unit tests for those use cases are included side by side with the components in the repositories. Integrated verification is performed through the comparison of the expected against the measured systemic impact of a new or modified component.

#### Sensitivity analyses and uncertainty quantification

Most Mouse Analog Mechanisms are inscribed with a probability distribution and executed according to Monte Carlo sampling followed by aggregation of results. Simultaneous, small changes (e.g., 5–10%) of several configuration values can offset each other and may produce no detectable change in measured events or outcomes. Thus, linear sensitivity studies are less informative and meaningful than complete location changes in the space of Analog configurations, as done for Fig 7A. A complication is that significant regions of Mouse Analog’s configuration space may be non-biomimetic. For example, 1) having more Sinusoid Segments in Zone 3 than in Zone 2 or having the probability of NAPQI formation in Zone 1 be greater than in Zone 3 is not biomimetic. Domain knowledge is used to select for analysis only in those regions of configuration space that are known to be, or are plausibly, biomimetic. We use batch sampling [29] of the space of configurations to identify small configuration subsets (such as the values and their zonation in Fig 4B) that are most influential for particular AILI attributes.

Some uncertainty is built into each Lobule. Sources include Monte Carlo variations in Sinusoid Segment dimensions and graph composition, probabilistic events, networking of some events, and small numbers of objects. Thus, no two Lobule simulations are the same and some phenomena measured during a single execution can exhibit large discontinuities. Consequently, experiments are comprised of 12 (Fig 7A) to 332 (Fig 6) Monte Carlo Lobule variants.

Scripts for analysis are essential components of incremental validation. They are checked into the repositories as experiment workflows. For each simulation, important event information, such as timing and location of Hepatocyte Deaths, are recorded along with a selection of other events influential in Hepatocyte Death patterns (e.g., zonation of GSH Depletion events). This record mimics possible experimental measurements and provides a sampling of the entire biomimetic Mechanism space. This information can be used to separate the space of feature configurations into regions which have achieved validation and which were falsified. Mappings between output variance, which is a consequence of inscribed stochasticity, and variance in wet-lab data provide a quantification of uncertainty.

## Supporting Information

S1 Table Configuration details for Mechanisms in Fig 5. (PDF)

S2 Table Values for Configurations used in generating results in Fig 7A and Supporting S2 Fig (PDF)

S3 Table Features of Mechanisms (PDF)

S1 Fig **Target Attributes: pharmacokinetic similarities** (A) Shown are similarities between pharmacokinetic profiles in Mouse Body and in plasma from Swiss Webster mice administered 400 mg/kg APAP by oral intubation as reported by Fischer et al. [16]. The Analog to wet-lab data mapping [25] assumes a direct correlation between APAP in Mouse Body and APAP per gram in tissue samples, including plasma. We imposed a stringent Similarity Criterion: measurements of APAP in Mouse Body (maps to APAP concentrations in plasma) must be within 1 standard deviation of the mouse data (vertical bars). The Mouse Analog and APAP dose are the same as in Figs 6 and 7. (B and C) We demonstrate characteristic dose-dependent pharmacokinetics by scaling results of Mouse Analog experiments to simulate reported dose-dependent pharmacokinetic data in rats. As in A, the Analog to wet-lab data mapping assumes a direct correlation between APAP in Mouse Body and concentration of APAP in plasma. Control is the APAP profile in Fig 6B (maps to 300 mg/kg in mice. The APAP plasma half-life of APAP in rats is approximately 3x-5x that in mice [40]. Using the median value of 4x, Control dose scales to approximately 75 mg/kg in rats. Profiles resulting from doses of 2x and 0.5x APAP objects should scale to about 150 mg/kg and 37.5 mg/kg, respectively, in rats. Plasma profiles in B are from Galinsky and Levy [38]; plasma profiles in C are from Hjelle and Klaassen [39].
(PNG)

S2 Fig **Additional details for selected variants in Fig 7A** These details are for the 20 variants shaded green in Fig 7A. Our working hypothesis is that 24-hour necrosis scores (same experiment) for same-strain mice that are within about 20% of each other can be judged experimentally indistinguishable. Total 24 hour Dead Hepatocytes for these 20 Mechanism variants are within 20% of MGNZ-Mechanism (vertical gray bar at #35). Note that the relative differences in bar heights for Zones 1–3 between MGNZ-Mechanism and other 20 variants. Those differences reflect differences in the causal cascades between MGNZ-Mechanism and those of the 20 variants. Differences among these analogs may map to differences among individual mice within the same wet-lab experiment. Near PP is defined as > 30 grid spaces from CV. Values for each variant configuration are provided in Supporting S2 Table.
(PNG)

S3 Fig **Configurations used in generating results in Fig 7A** (A) These three Mechanism features are the same as those in Fig 4A. Black lines identify a location specific feature configuration for MGNZ-Mechanism. Each red line identifies an alternative feature configuration used in one or more of the variants in Fig 7A. The particular instance for each use of alternative feature configurations is listed in Supporting S3 Table. Probabilities are per time step (maps to 1 second). B) These two Mechanism features are the same as those in Fig 4B. The meaning of black and red feature configurations is as stated in A. (C) Each red line is an alternative configuration used by one or more of the Mechanism variants in Fig 7A and in Supporting S4 Fig. Sampling the space of feature configurations and their settings differs fundamentally from sampling parameter space during a sensitivity analysis for a differential equation based model. That is in part because, for current use cases, large regions of the space of Mouse Analog feature configurations yield Mouse Analog variants that are not biomimetic. For example, specifying that the probability of an APAP Metabolism event is independent of Lobule location is not biomimetic because no supportive evidence has been reported. For the same reason, it is not biomimetic to specify that the probability of APAP metabolism decreases PP to CV. A feature configuration that results in NAPQI being the primary Metabolite would also be non-biomimetic: all the available data indicate that it is a minor metabolite. See *Sensitivity analyses and uncertainty quantification* subsection for additional explanations. The sampling of the space of Mouse Analog configuration settings had two objectives. 1) Identify small sets of biologically plausible changes in configuration settings that measurably alter the Mechanism (as evidenced by differences in Hepatocyte Death zonation), but keep the change in total Hepatocyte Deaths within 20% of those for MGNZ-Mechanism: changes in the configurations selected tend to cancel each other. See Supporting S4 Fig for examples. 2) Identify other small sets of biologically plausible changes in configuration settings that appear somewhat synergistic when combined: small changes in configurations alone are relatively inconsequential, but when combined generate larger changes in total Hepatocyte Deaths. Examples are left and right of the green bars in Fig 7A.
(PNG)

S4 Fig **Biomimetic Lobule gradient** Illustrated is the non-linear PP to CV gradient [13] referred to in Results. It maps to measures of one or more common blood attributes, such as pO_2_ [35].
(PNG)

S5 Fig **Target Attribute: Measurable Dead Hepatocytes** (A) Illustrated is the strategy used described in Materials and Methods to identify a *Death Delay* rule. To specify the *Death Delay* feature, we needed a Target Attribute estimate for percent total necrosis as a function of time post-dose. The shaded area is the estimate provided coauthor Kaplowitz. The Similarity Criterion is that cumulative total Hepatocyte Deaths fall within the shaded area. Following multiple Iterative Refinement Protocol cycles, we identified the two *Death Delay* rules shown. The orange curve results from specifying that a Hepatocyte *Death Delay* is determined by a pseudo-random draw from a normal distribution having a mean of mean of 7.2 hours with standard deviation of 4.1 hours. The blue curve specifying *Death Delay* is determined by a pseudo-random draw from uniform [1.2, 12] hours. We use the latter in generating all results described in Results because it is the more parsimonious option. (B) Shown are Cumulative Dead Hepatocytes for the MGNZ-Mechanism experiment in Fig 7 for the first 6 hours post-dose. The axis specifies the percent of total Hepatocytes.
(PNG)
