# Supplementary material for: Competing Mechanistic Hypotheses of Acetaminophen-Induced Hepatotoxicity Challenged by Virtual Experiments
Source: PLoS Comput Biol. 2016 Dec 16;12(12):e1005253. doi: 10.1371/journal.pcbi.1005253 (PMC5161305; doi:10.1371/journal.pcbi.1005253)
Supplement: S1 Table — (PDF) [file pcbi.1005253.s001.pdf]

Supporting S1 Table | Configuration details for Mechanisms in Fig. 5

Feature	Description	Defaults	Type	Range	Mechanisms			
					NZ-	GNZ-	MNZ-	MGNZ-
**APAP Metabolism**								
rxnProb <PP, CV>	APAP metabolism probability	<0.35,0.95>	real	[0, 1]				
APAP → G	APAP glucuronidiation fraction	<0.33, 0.05>	real	[0, 1]				
APAP → S	APAP sulphation fraction	<0.33, 0.05>	real	[0, 1]				
APAP → N	APAP to NAPQI fraction	<0.34, 0.9>	real	[0, 1]				
**Damage Production**								
rxnProb <PP, CV>	NAPQI reaction probability	<0.5, 0.5>	real	[0, 1]				
N → nMD	NAPQI to non-Mitochondrial damage fraction	<0.5, 0.5>	real	[0, 1]				
N → MitoD	NAPQI to Mitochondrial damage fraction	<0.5, 0.5>	real	[0, 1]				
MitoD Amplify	Mitochondrial damage amplification	TRUE	boolean	TRUE or FALSE				
**Damage Mitigation**								
nMD rxnProb <PP, CV>	Non-Mitochondrial mitigation probability	<0.2, 0.8>	real	[0, 1]				
nMD → R	Non-Mitochondrial mitigation fraction	<1.0, 1.0>	real	[0, 1]				
nMD rxnProb Gradient	non-Mitochondrial mitigation gradient shape	linear	string	linear or sigmoidal				
MitoD rxnProb <PP, CV>	Mitochondrial damage mitigation probability	<0.9, 0.0>	real	[0, 1]	<0.6, 0.6>	<0.6, 0.6>	<0.9, 0.0>	<0.9, 0.0>
MitoD rxnProb Gradient	Mitochondrial mitigation gradient shape	sigmoidal	string	linear or sigmoidal	linear, constant	linear, constant	sigmoidal	sigmoidal
MitoD → R	Mitochondrial mitigation fraction	<1.0, 1.0>	real	[0, 1]				
**GSH Depletion & Cell Death**								
gshDepletionRange	GSH depletion threshold	<5.0, 0.0>	real	[0,∞)	<3.0, 3.0>	<5.0, 0.0>	<3.0, 3.0>	<5.0, 0.0>
deathRange <PP, CV>	MitoD damage for cell death trigger threshold	<6, 6>	integer	[0, ∞)				
deathDelayMin	minimum time until cell death	540	integer	[0, ∞)				
deathDelayMax	maximum time until cell death	6600	integer	[0, ∞)				
