# Supplementary material for: Competing Mechanistic Hypotheses of Acetaminophen-Induced Hepatotoxicity Challenged by Virtual Experiments
Source: PLoS Comput Biol. 2016 Dec 16;12(12):e1005253. doi: 10.1371/journal.pcbi.1005253 (PMC5161305; doi:10.1371/journal.pcbi.1005253)
Supplement: S2 Table — (PDF) [file pcbi.1005253.s002.pdf]

Supporting S2 Table | Configuration settings used in for Fig. 7A and S2 Figure. See panel descriptions in Supporting S3 Figure.

Black: MGNZ-Meachanism Configuration Settings; Red: Variant Configuration Settings

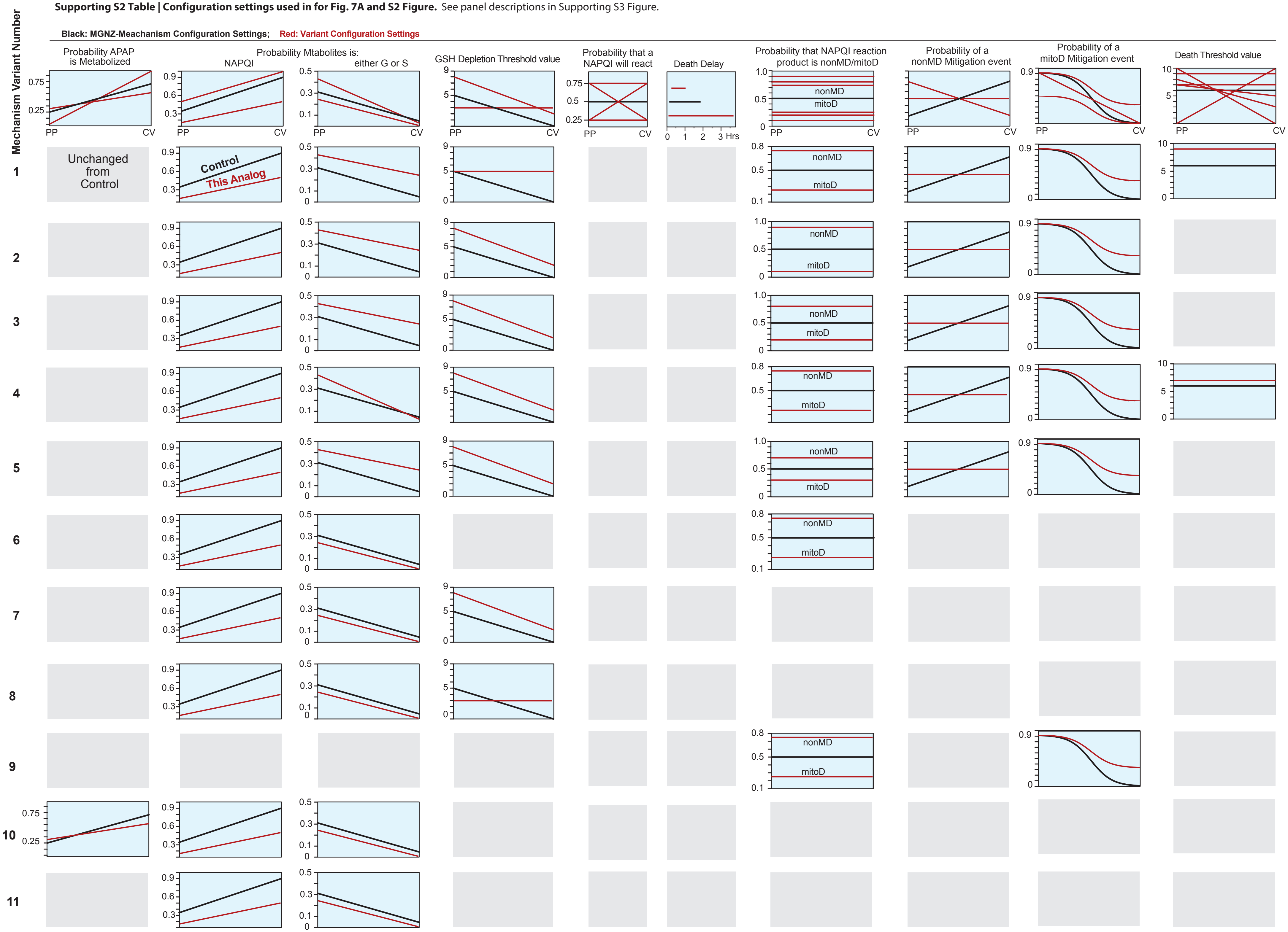

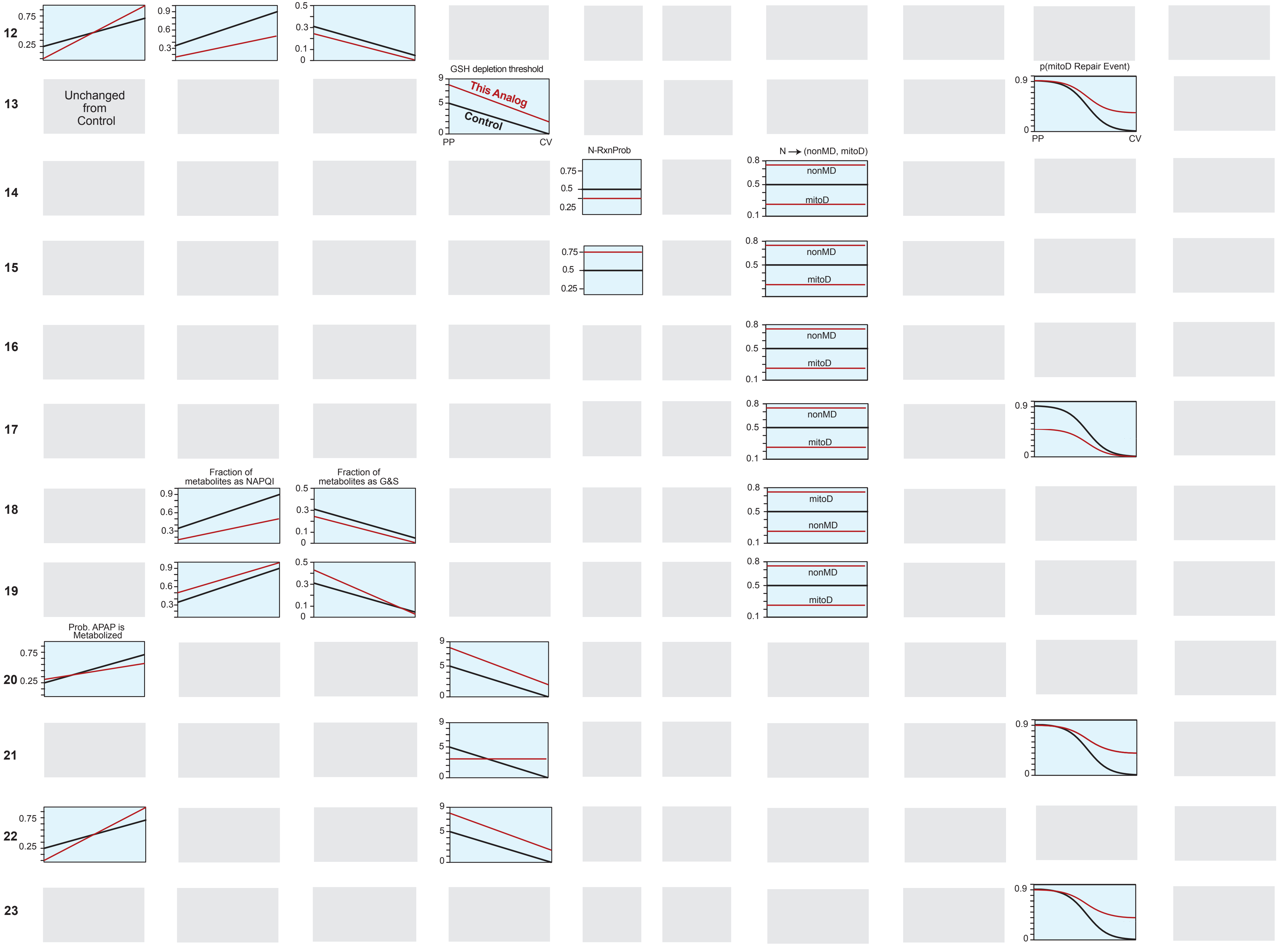

12
13
14
15
16
17
18
19
20
21
22
23
Unchanged from Control
GSH depletion threshold
This Analog
Control
PP
CV
p(mitoD Repair Event)
N-RxnProb
N → (nonMD, mitoD)
nonMD
mitoD
Fraction of metabolites as NAPQI
Fraction of metabolites as G&S
Prob. APAP is Metabolized

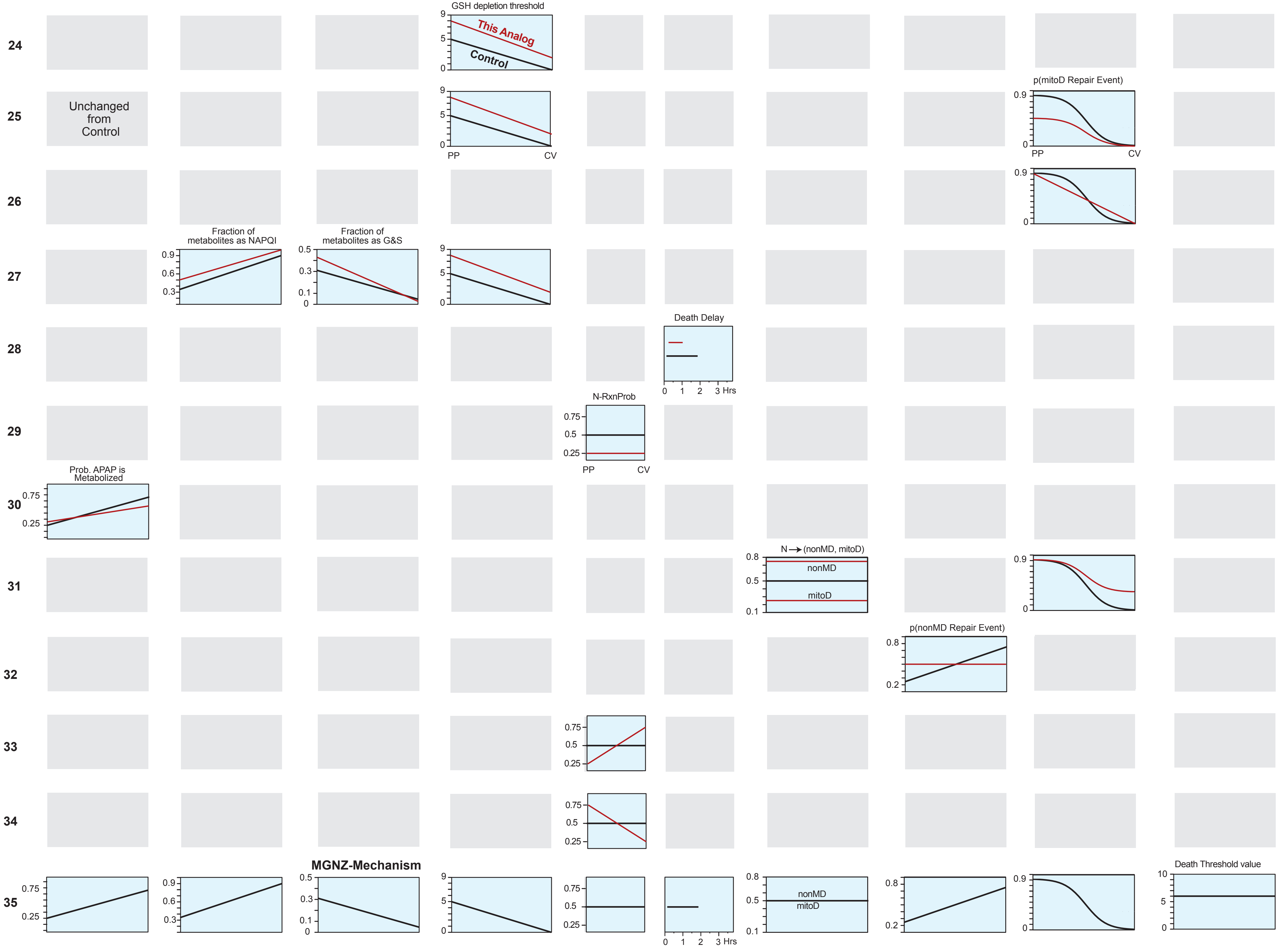
24
25
26
27
28
29
30
31
32
33
34
35
GSH depletion threshold
This Analog
Control
Unchanged from Control
PP
CV
p(mitoD Repair Event)
Fraction of metabolites as NAPQI
Fraction of metabolites as G&S
Death Delay
0 1 2 3 Hrs
N-RxnProb
Prob. APAP is Metabolized
N → (nonMD, mitoD)
nonMD
mitoD
p(nonMD Repair Event)
MGNZ-Mechanism
Death Threshold value

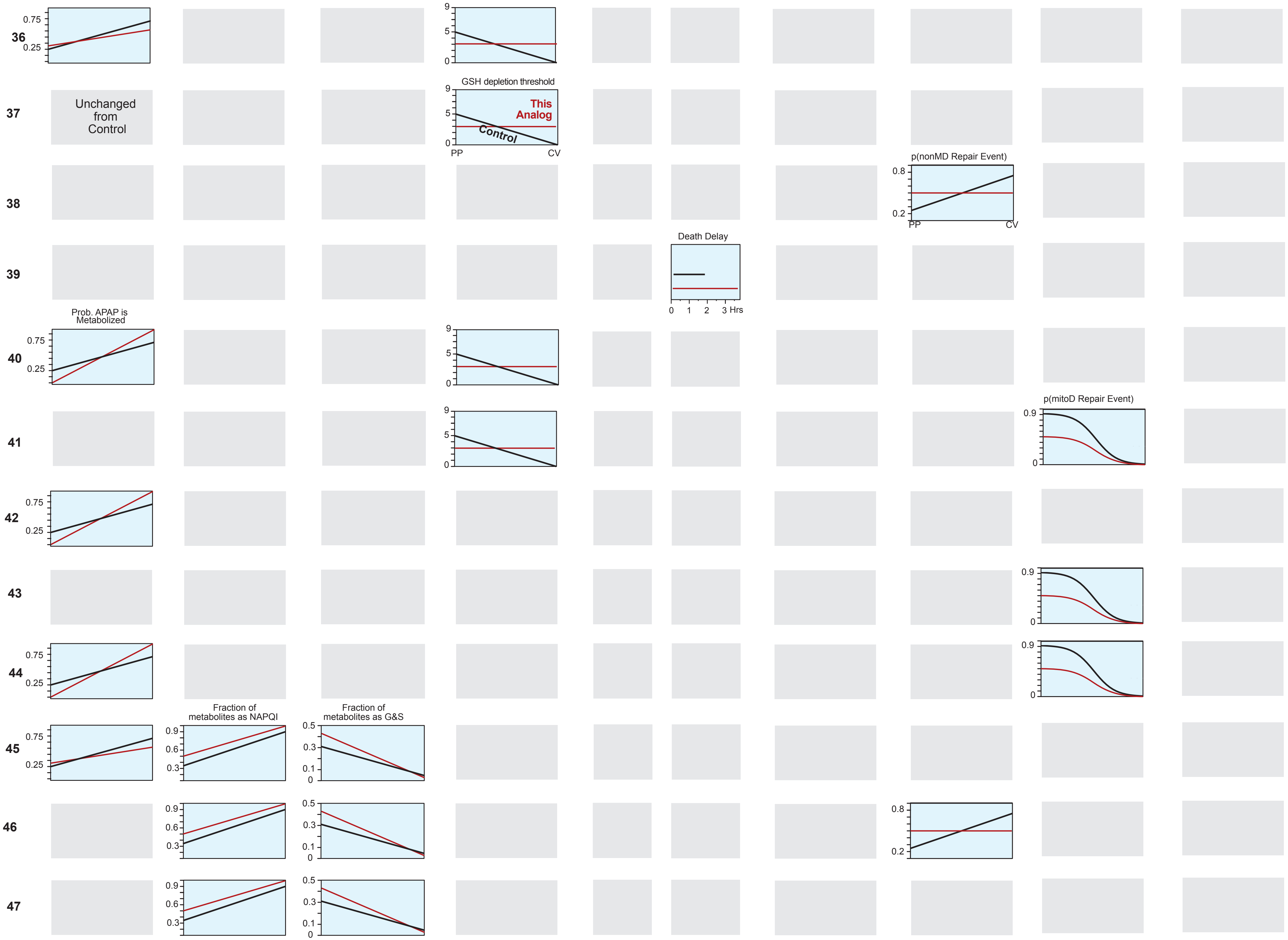

36
37
38
39
40
41
42
43
44
45
46
47
Unchanged from Control
GSH depletion threshold
This Analog
Control
PP
CV
p(nonMD Repair Event)
Death Delay
0 1 2 3 Hrs
Prob. APAP is Metabolized
p(mitoD Repair Event)
Fraction of metabolites as NAPQI
Fraction of metabolites as G&S

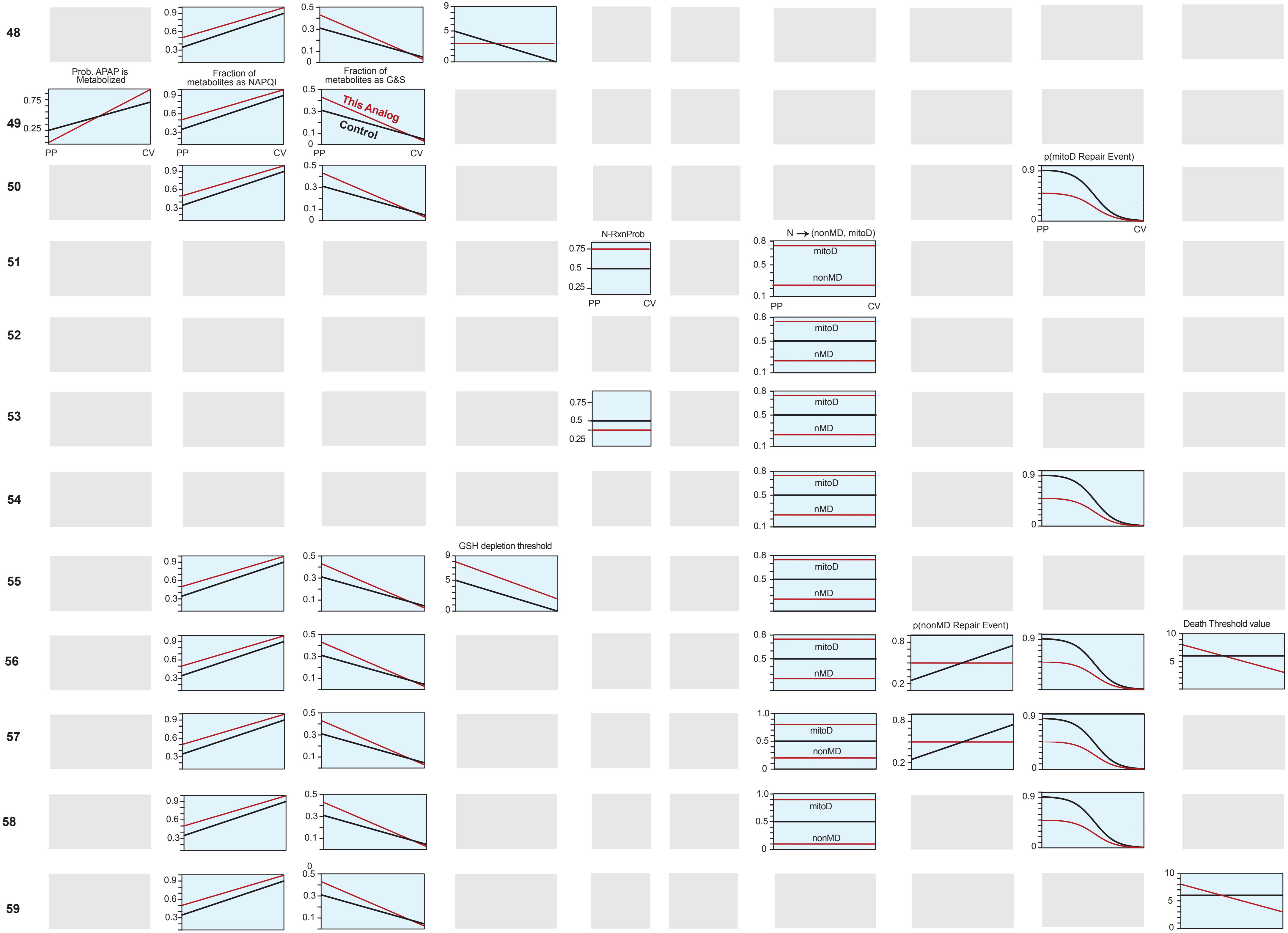
48
49
50
51
52
53
54
55
56
57
58
59
Prob. APAP is Metabolized
Fraction of metabolites as NAPQI
Fraction of metabolites as G&S
This Analog
Control
PP
CV
p(mitoD Repair Event)
N-RxnProb
N → (nonMD, mitoD)
mitoD
nonMD
nMD
GSH depletion threshold
p(nonMD Repair Event)
Death Threshold value

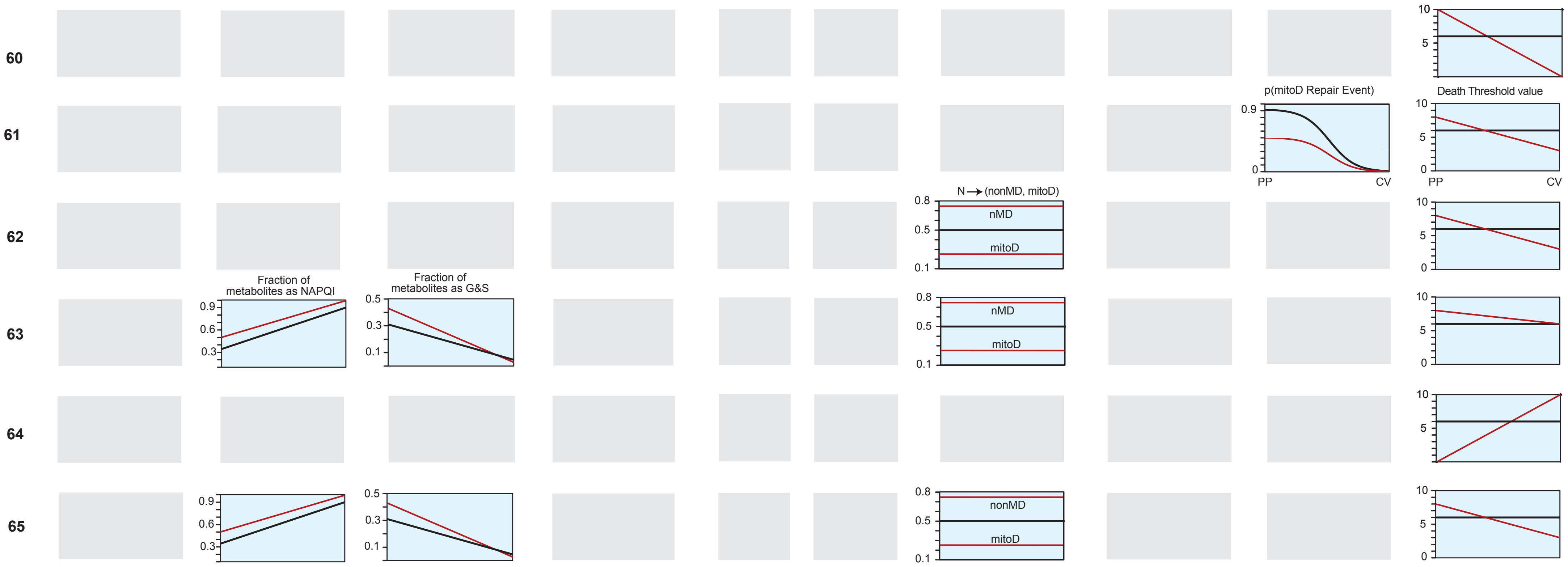

60
61
62
63
64
65
p(mitoD Repair Event)
Death Threshold value
N → (nonMD, mitoD)
nMD
mitoD
nonMD
Fraction of metabolites as NAPQI
Fraction of metabolites as G&S
PP
CV
