# Supplementary material for: Competing Mechanistic Hypotheses of Acetaminophen-Induced Hepatotoxicity Challenged by Virtual Experiments
Source: PLoS Comput Biol. 2016 Dec 16;12(12):e1005253. doi: 10.1371/journal.pcbi.1005253 (PMC5161305; doi:10.1371/journal.pcbi.1005253)
Supplement: S3 Table — (PDF) [file pcbi.1005253.s003.pdf]

Supporting S3 Table | Darden's [24] **Features of Mechanisms**

**As defined by Darden**	**Mouse Analog Examples**
Phenomenon	Necrosis begins close to CV and progresses outward
Components	
Entities and activities	Hepatocyte, mitoD, Metabolism
Modules	SS, "DeathHandler", "ReactionHandler"
Spatial arrangement of components	
Localization	Hepatocytes at a certain distance from CV
Structure	Lobule's directed graph, Sinusoid Segments
Orientation	Flow is PP to CV
Connectivity	Connectivity of a few SS from Zone 1 to Zone 3
Compartmentalization	GSH depletion events are Hepatocyte specific
Temporal aspects of components	
Order	A Binding event precedes Metabolism;<br>A trigger event precedes Death
Rate	Simulated IP absorption of APAP;<br>mitoD Mitigation
Duration	*Death Delay*
Frequency	Time steps
Contextual locations	
Location within a hierarchy	Hepatocyte within SS within Lobule within Mouse Analog
Location within a series	Toxicity Phase, Fig. 3B
