# Supplementary figures and images for: Competing Mechanistic Hypotheses of Acetaminophen-Induced Hepatotoxicity Challenged by Virtual Experiments
Source: PLoS Comput Biol. 2016 Dec 16;12(12):e1005253. doi: 10.1371/journal.pcbi.1005253 (PMC5161305; doi:10.1371/journal.pcbi.1005253)

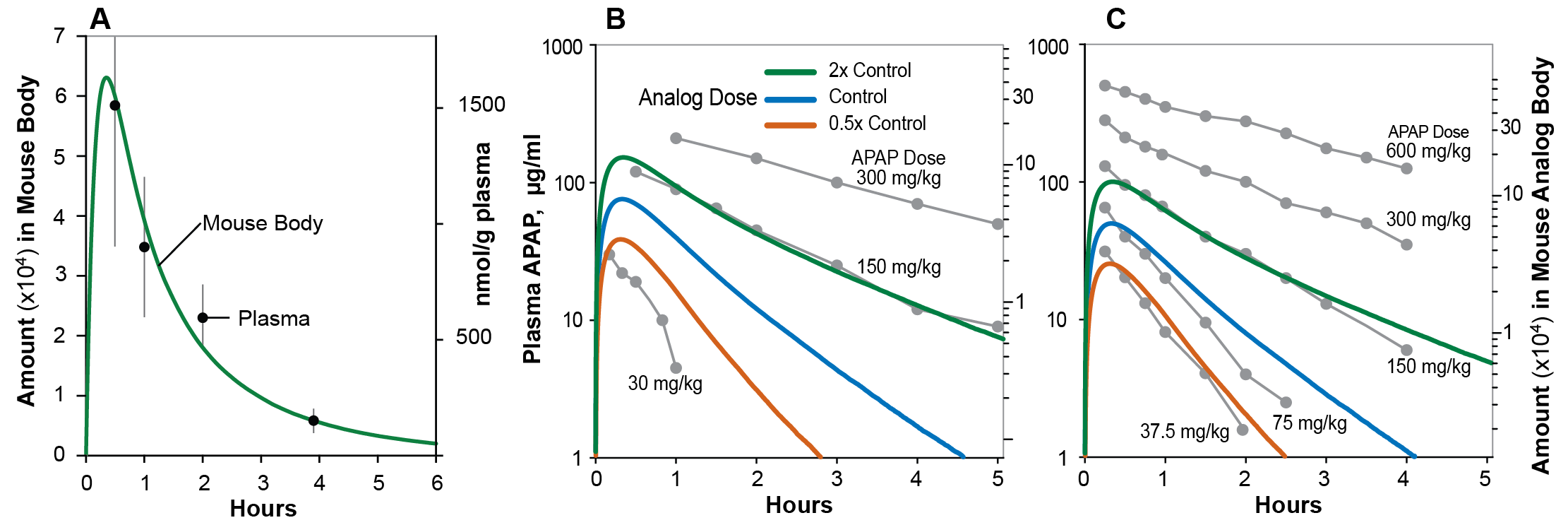

Supplement: S1 Fig — Target Attributes: pharmacokinetic similarities (A) Shown are similarities between pharmacokinetic profiles in Mouse Body and in plasma from Swiss Webster mice administered 400 mg/kg APAP by oral intubation as reported by Fischer et al. [16]. The Analog to wet-lab data mapping [25] assumes a direct correlation between APAP in Mouse Body and APAP per gram in tissue samples, including plasma. We imposed a stringent Similarity Criterion: measurements of APAP in Mouse Body (maps to APAP concentrations in plasma) must be within 1 standard deviation of the mouse data (vertical bars). The Mouse Analog and APAP dose are the same as in Figs 6 and 7. (B and C) We demonstrate characteristic dose-dependent pharmacokinetics by scaling results of Mouse Analog experiments to simulate reported dose-dependent pharmacokinetic data in rats. As in A, the Analog to wet-lab data mapping assumes a direct correlation between APAP in Mouse Body and concentration of APAP in plasma. Control is the APAP profile in Fig 6B (maps to 300 mg/kg in mice. The APAP plasma half-life of APAP in rats is approximately 3x-5x that in mice [40]. Using the median value of 4x, Control dose scales to approximately 75 mg/kg in rats. Profiles resulting from doses of 2x and 0.5x APAP objects should scale to about 150 mg/kg and 37.5 mg/kg, respectively, in rats. Plasma profiles in B are from Galinsky and Levy [38]; plasma profiles in C are from Hjelle and Klaassen [39]. (PNG) [file pcbi.1005253.s004.png]

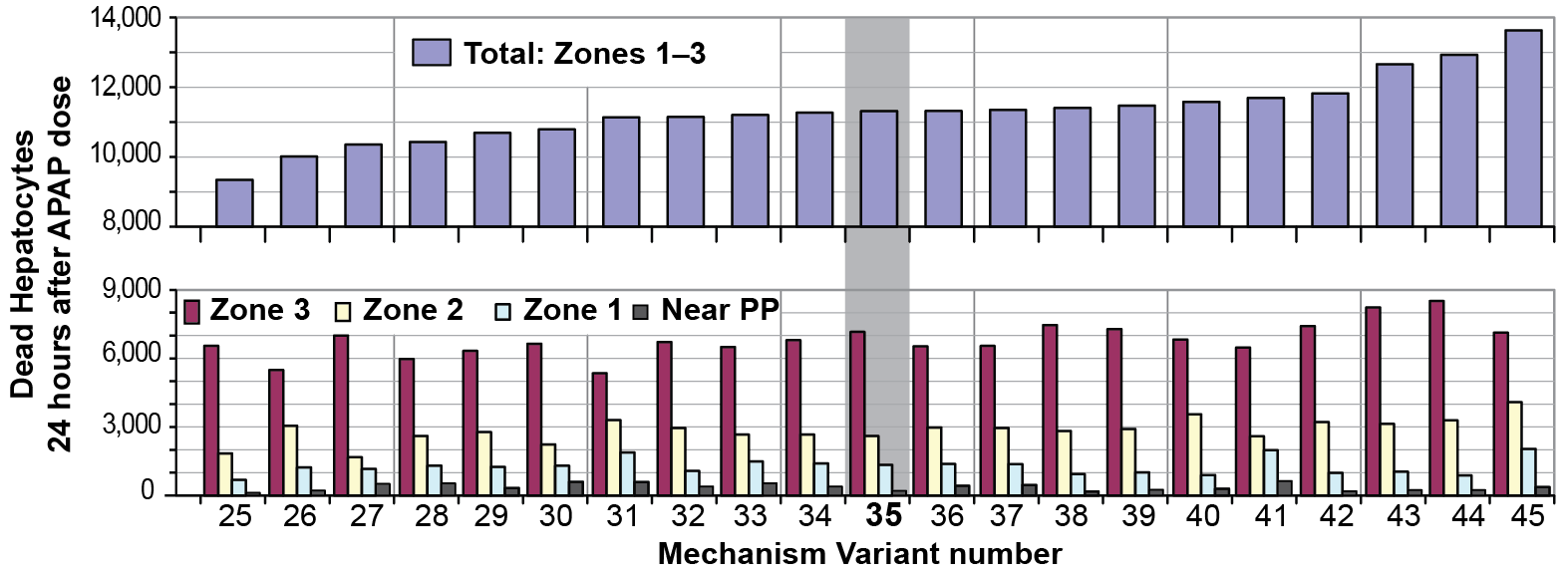

Supplement: S2 Fig — Additional details for selected variants in Fig 7A These details are for the 20 variants shaded green in Fig 7A. Our working hypothesis is that 24-hour necrosis scores (same experiment) for same-strain mice that are within about 20% of each other can be judged experimentally indistinguishable. Total 24 hour Dead Hepatocytes for these 20 Mechanism variants are within 20% of MGNZ-Mechanism (vertical gray bar at #35). Note that the relative differences in bar heights for Zones 1–3 between MGNZ-Mechanism and other 20 variants. Those differences reflect differences in the causal cascades between MGNZ-Mechanism and those of the 20 variants. Differences among these analogs may map to differences among individual mice within the same wet-lab experiment. Near PP is defined as > 30 grid spaces from CV. Values for each variant configuration are provided in Supporting S2 Table. (PNG) [file pcbi.1005253.s005.png]

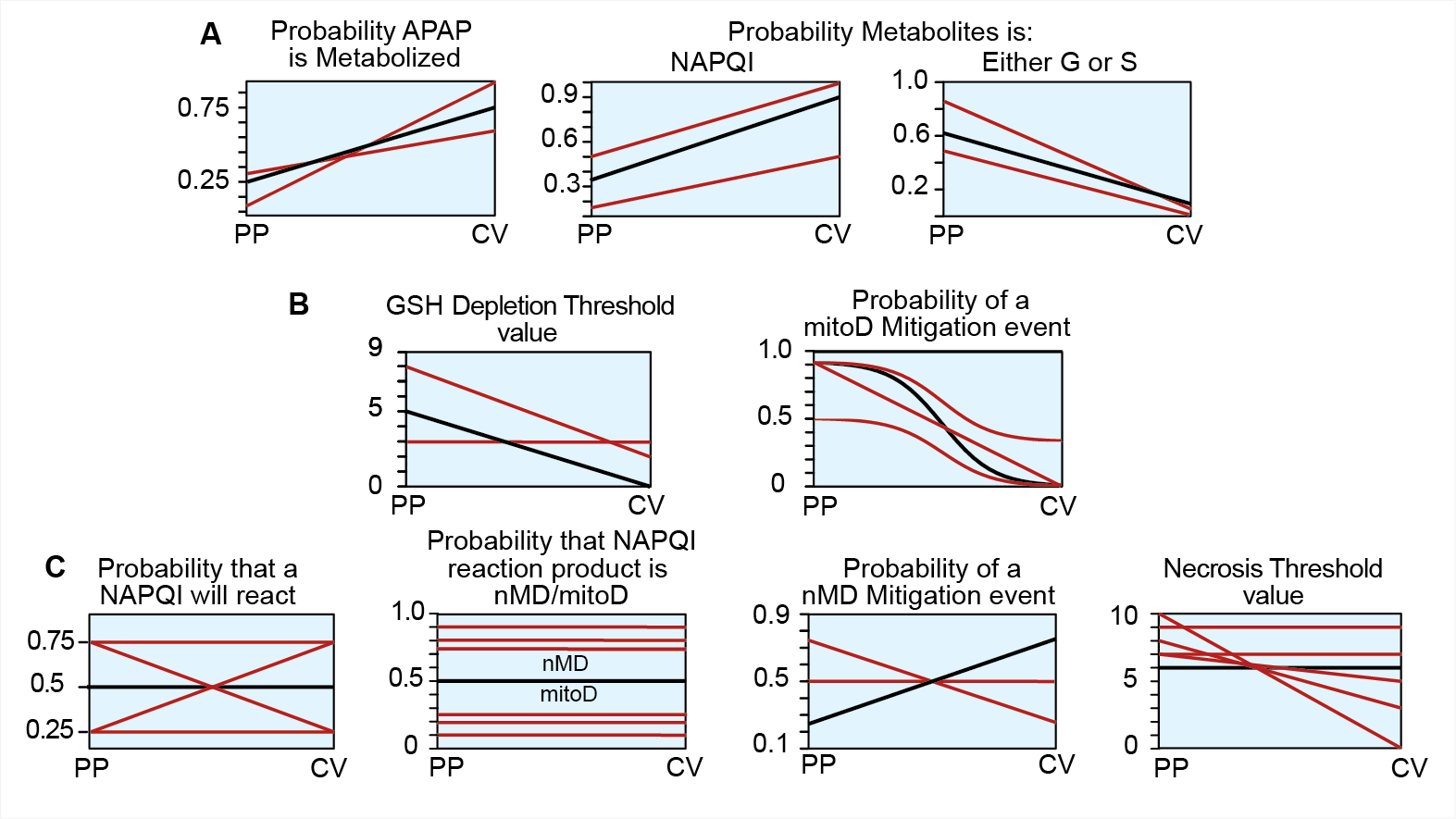

Supplement: S3 Fig — Configurations used in generating results in Fig 7A (A) These three Mechanism features are the same as those in Fig 4A. Black lines identify a location specific feature configuration for MGNZ-Mechanism. Each red line identifies an alternative feature configuration used in one or more of the variants in Fig 7A. The particular instance for each use of alternative feature configurations is listed in Supporting S3 Table. Probabilities are per time step (maps to 1 second). B) These two Mechanism features are the same as those in Fig 4B. The meaning of black and red feature configurations is as stated in A. (C) Each red line is an alternative configuration used by one or more of the Mechanism variants in Fig 7A and in Supporting S4 Fig. Sampling the space of feature configurations and their settings differs fundamentally from sampling parameter space during a sensitivity analysis for a differential equation based model. That is in part because, for current use cases, large regions of the space of Mouse Analog feature configurations yield Mouse Analog variants that are not biomimetic. For example, specifying that the probability of an APAP Metabolism event is independent of Lobule location is not biomimetic because no supportive evidence has been reported. For the same reason, it is not biomimetic to specify that the probability of APAP metabolism decreases PP to CV. A feature configuration that results in NAPQI being the primary Metabolite would also be non-biomimetic: all the available data indicate that it is a minor metabolite. See Sensitivity analyses and uncertainty quantification subsection for additional explanations. The sampling of the space of Mouse Analog configuration settings had two objectives. 1) Identify small sets of biologically plausible changes in configuration settings that measurably alter the Mechanism (as evidenced by differences in Hepatocyte Death zonation), but keep the change in total Hepatocyte Deaths within 20% of those for MGNZ-Mechanism: ch [file pcbi.1005253.s006.png]

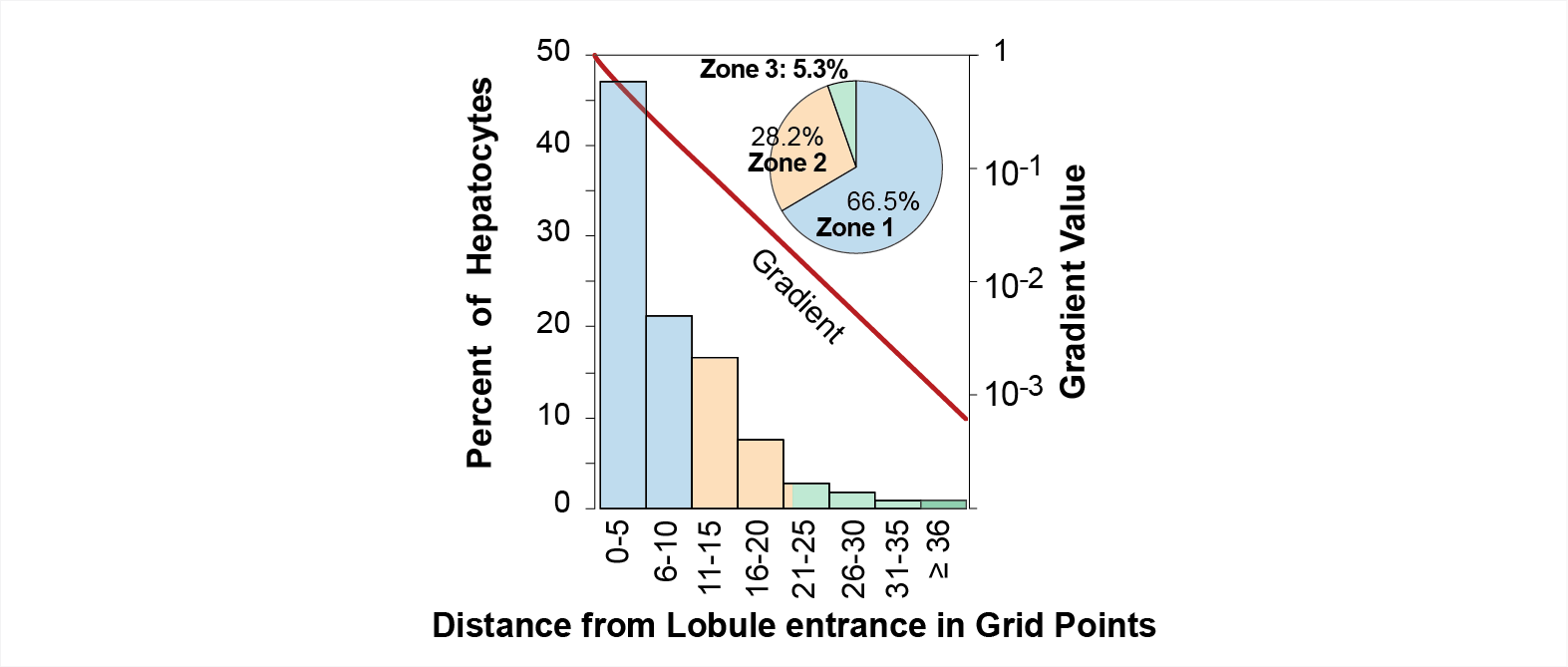

Supplement: S4 Fig — Biomimetic Lobule gradient Illustrated is the non-linear PP to CV gradient [13] referred to in Results. It maps to measures of one or more common blood attributes, such as pO2 [35]. (PNG) [file pcbi.1005253.s007.png]

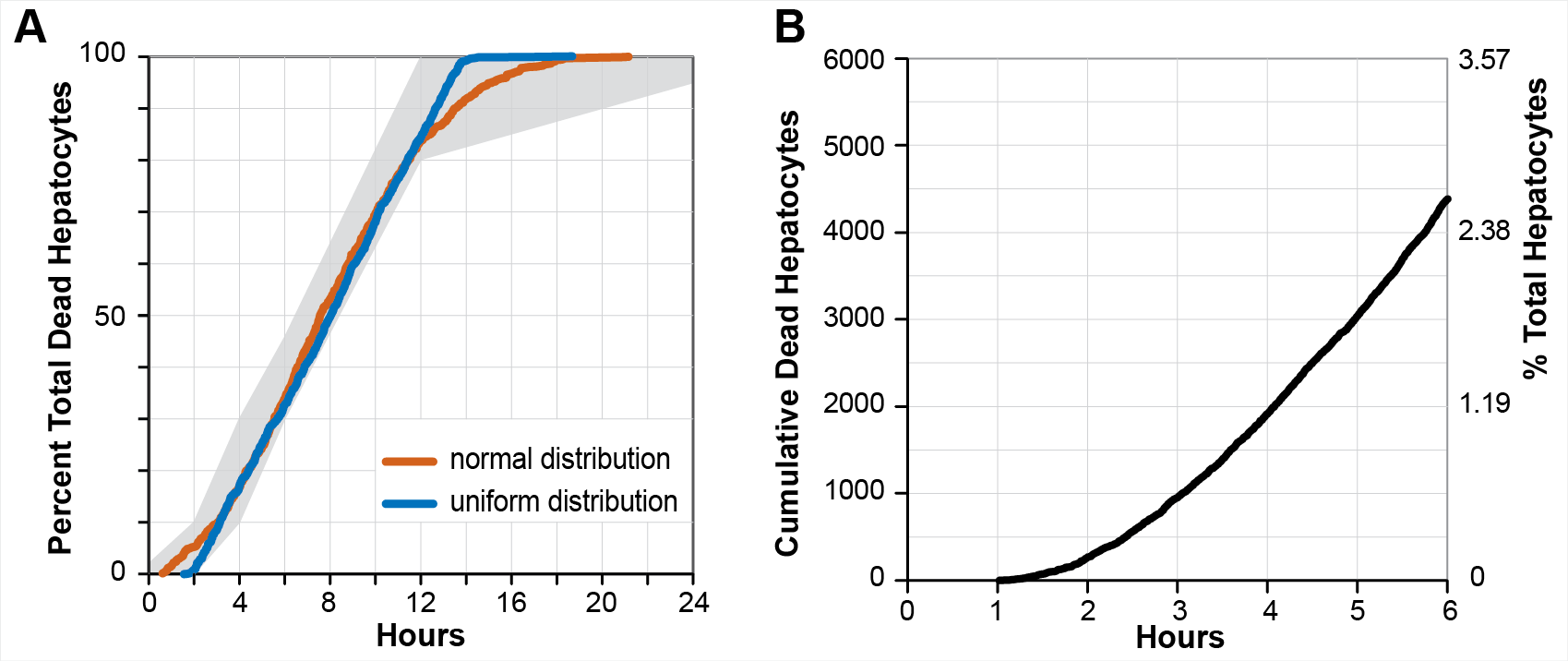

Supplement: S5 Fig — Target Attribute: Measurable Dead Hepatocytes (A) Illustrated is the strategy used described in Materials and Methods to identify a Death Delay rule. To specify the Death Delay feature, we needed a Target Attribute estimate for percent total necrosis as a function of time post-dose. The shaded area is the estimate provided coauthor Kaplowitz. The Similarity Criterion is that cumulative total Hepatocyte Deaths fall within the shaded area. Following multiple Iterative Refinement Protocol cycles, we identified the two Death Delay rules shown. The orange curve results from specifying that a Hepatocyte Death Delay is determined by a pseudo-random draw from a normal distribution having a mean of mean of 7.2 hours with standard deviation of 4.1 hours. The blue curve specifying Death Delay is determined by a pseudo-random draw from uniform [1.2, 12] hours. We use the latter in generating all results described in Results because it is the more parsimonious option. (B) Shown are Cumulative Dead Hepatocytes for the MGNZ-Mechanism experiment in Fig 7 for the first 6 hours post-dose. The axis specifies the percent of total Hepatocytes. (PNG) [file pcbi.1005253.s008.png]
